# Nonlinear reversal of photoexcitation on the attosecond time scale improves ultrafast X-ray diffraction images

**DOI:** 10.1038/s41467-026-75969-8

**Published:** 2026-07-28

**Authors:** Anatoli Ulmer, Phay J. Ho, Bruno Langbehn, Stephan Kuschel, Linos Hecht, Razib Obaid, Simon Dold, Taran Driver, Joseph Duris, Ming-Fu Lin, David Cesar, Paris Franz, Zhaoheng Guo, Philip A. Hart, Andrei Kamalov, Kirk A. Larsen, Xiang Li, Michael Meyer, Kazutaka Nakahara, Robert G. Radloff, River Robles, Lara Rönnebeck, Nick Sudar, Adam M. Summers, Linda Young, Peter Walter, James P. Cryan, Christoph Bostedt, Daniela Rupp, Agostino Marinelli, Tais Gorkhover

**Affiliations:** 1 CFEL, Universität Hamburg, Hamburg, Germany; 2 Argonne National Laboratory, Argonne, IL USA; 3 Institute of Optics and Atomic Physics, Technische Universität Berlin, Berlin, Germany; 4 Institute of Nuclear Physics, Technical University Darmstadt, Darmstadt, Germany; 5 ETH Zurich, Laboratory of Solid State Physics, Zurich, Switzerland; 6 SLAC National Accelerator Laboratory, Menlo Park, CA USA; 7 European XFEL, Schenefeld, Germany; 8 Stanford PULSE Institute, SLAC National Accelerator Laboratory, Menlo Park, CA USA; 9 Department of Applied Physics, Stanford University, Stanford, CA USA; 10 PSI, Paul-Scherrer-Institute, Villigen, Switzerland; 11 Laboratory for Ultrafast X-ray Sciences, Institute of Chemical Sciences and Engineering (LUXS), École Polytechnique Fédérale de Lausanne (EPFL), Lausanne, Switzerland

**Keywords:** X-rays, Nonlinear optics

## Abstract

The complex refractive index of a material governs its light-matter interactions, with intense light fields enabling tailored nonlinear optical responses. In the X-ray regime, rapid photoionization limits the potential of nonlinear techniques by inducing irreversible electronic damage. Here we demonstrate that intense, sub-femtosecond X-ray pulses, shorter than typical Auger decay times, can partially reverse photoexcitation via stimulated emission near atomic resonances. By analyzing thousands of coherent diffraction patterns and ion spectra from neon nanoparticles exposed to sub-fs and 15-fs pulses, we observe enhanced X-ray diffraction alongside reduced energy absorption for sub-fs pulses. Theoretical modeling attributes this to dynamics akin to Rabi flopping that prolong the lifetime of resonant states and suppress electronic bleaching. These findings suggest that ultrashort, intense X-ray pulses enable active control of X-ray refractive index and damage pathways, opening avenues for improved high-resolution imaging and nonlinear spectroscopy in complex nanoscale systems.

## Abstract (web summary)

Despite recent successes in X-ray imaging due to ultrashort XFEL pulses, current techniques are limited by the strong-field photoionisation damaging the sample structure. Here, the authors demonstrate reversion of ionisation processes on Ne nanoparticles under subfemtosecond-length pulses.

## Introduction

Non-linear optics has enabled groundbreaking technologies ranging from imaging^1^ and spectroscopy^2^ to quantum communication^3^. In the X-ray regime, sample degradation through photoionization has been the ultimate limit for applications requiring high X-ray intensities^4–9^. A prominent example is ultrafast, high-resolution X-ray imaging. Achieving higher contrast and improved spatial resolution generally requires increasing the number of X-ray photons per unit area^10^. However, higher X-ray fluence inevitably increases photoionization rates, compromising the structural integrity of the sample. The maximum tolerable number of X-ray photons during the exposure sets a fundamental limit on the structural information content of the image. This constraint persists even in advanced approaches such as diffraction-before-destruction imaging^4,11–13^. Here, intense X-ray free-electron laser (FEL) flashes as short as a few femtoseconds outrun the atomic structure degradation and provide unique insights into non-equilibrium dynamics in molecules and other nanoscale specimens with unprecedented temporal and spatial precision^14–24^. A key limitation of this approach remains the electronic bleaching of the sample^4,10,25–27^. Intense X-ray pulses rapidly deplete electronic ground states within femtoseconds through excitation, photoionization, and subsequent relaxation cascades. In extreme cases, many electrons are removed from the parent ions and atoms become effectively transparent to the probe before significant atomic motion occurs. Such electronic bleaching or damage is regarded as the ultimate limit for the diffraction signal in high scattering angles and thus, to the spatial resolution in X-ray diffraction imaging. Electron bleaching also affects the sensitivity and accuracy in spectroscopic studies, especially for site-specific nonlinear schemes in complex specimens^12,23,25,27–34^.

Over the past decade, multiple studies spanning the XUV to the hard X-ray regime have shown that nonlinear effects, such as transient resonances, can substantially accelerate photoionization and, in consequence, electronic structure degradation^17,19,35–37^. When these resonances occur ions formed by X-rays can absorb much more strongly than neutral atoms. In a previous study on Xe nanoparticles, we have demonstrated that transient resonances can amplify soft X-ray small angle diffraction obtained with 10 fs short pulses beyond reported literature values^38^ before the onset of atomic structural disintegration^39^. However, we have also observed that transient resonances significantly accelerate electronic damage and the sample destruction due to increased absorption cross-sections. Thus, longer exposures (> 100 fs) resulted in a significant reduction of the scattering cross-section. This observation is consistent with earlier work on lighter-element nanoparticles (e.g., sucrose nanospheres), where 150-fs pulses led to reduced diffraction yield and accelerated structural degradation, both linked to transient resonances in oxygen ions^32^. Collectively, these findings established the prevailing view that the principal advantage of shorter pulses simply lies in their ability to outrun atomic motion. While transient resonances may increase scattering cross-sections in short-lived electronic states, these resonances accelerate electronic bleaching, which remains largely irreversible.

Here, we demonstrate that newly available intense sub-femtosecond FEL pulses fundamentally challenge this picture. They provide access to a transiently resonant regime in which stimulated emission can be exploited, enabling reversal of photoabsorption while preserving enhanced scattering cross-sections at extreme X-ray power densities approaching 10^20^ W/cm^2^. Rather than merely outrunning damage, our results point to active control of the refractive index and damage in dense and highly excited nanosystems.

The advent of high peak power TW 300–500 attosecond short X-ray pulses provides an interesting route to control electronic damage mechanisms^40–43^. Short, sub-fs, intense X-ray bursts are faster than relaxation processes such as Auger decay times of light elements, which are around 2–3 fs. The absence of dissipation cascades has a strong effect on the probability and significance of stimulated emission^33,34,44^. In our study, we show that the brightness of X-ray diffraction images from Ne nanoparticles can be enhanced using intense sub-femtosecond pulses tuned near the Ne K-edge, where stimulated emission partially reverses photoexcitation.

We compare sub-femtosecond exposures with snapshots recorded using 15-fs FEL pulses. Both pulse durations are sufficiently short to outrun atomic structure disintegration on the few-nanometer scale. First, we find that transient resonances enhance the scattering cross-section beyond neutral Ne values for both pulse durations and similar fluences, albeit at different photon energies. Second, sub-fs pulses deposit less total energy into the sample than 15-fs pulses, while yielding brighter diffraction at selected photon energies. Third, our simulations indicate that sub-fs X-ray pulses enhance the influence of stimulated emission, which substantially increases image brightness near the Ne K-edge while decreasing the total absorbed energy. Stimulated emission drives previously excited electrons back into the original transient resonant state, which is associated with an enhanced scattering cross-section.

Moreover, the diffraction yield from bound electrons which are directly linked to the position of ions is significantly higher for sub-fs exposures. In contrast, 15-fs pulses promote the formation of a hot, dense nanoplasma: although the ionic framework remains effectively frozen, many electrons contributing to the image become delocalized. Therefore, sub-fs pulses produce diffraction patterns that more closely represent the pristine sample, by limiting energy deposition and electronic damage.

## Results

### Single-shot FEL diffraction of isolated Ne nanoparticles

In our study, we explore the scattering cross-sections and X-ray absorption yields of individual Ne nanoparticles as a function of FEL pulse parameters such as FEL photon energy *ℏω*, FEL fluence and pulse duration *τ* (see Methods). For *τ* ≈ 0.3 fs to 0.5 fs, we have collected thousands of X-ray diffraction patterns and simultaneously recorded ion spectra at X-ray photon energies tuned to the vicinity of the Ne K-edge at 870 eV. We have also recorded a reference data set with *τ* ≈ 15 fs, which outruns ionic structure damage on the nanometer scale, but reflects the impact of electronic bleaching processes such as Auger decay and collisional ionization during nanoplasma formation^17,18,21,45^. The X-ray fluence was similar for both pulse durations. The average pulse energy for 15 fs pulses was 110 *μ*J and for sub-fs 60 *μ*J. The pulse energy distribution for sub-fs pulses was skewed towards higher pulse energies. In order to match those intense pulses, we chose to slightly increase the average pulses energy of the 15 fs pulses (see Table I and Suppl. Fig. 4 for details).

**Fig. 1:**
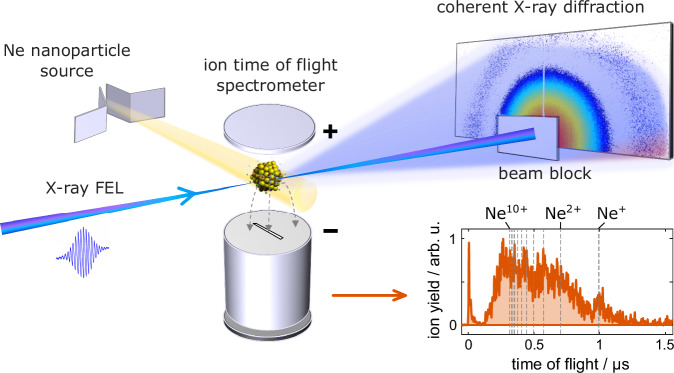
Experimental setup scheme. Individual Ne nanoparticles from a pulsed and cooled supersonic expansion Ne gas source are injected into the path of focused X-ray FEL flashes with different pulse lengths. Coherently diffracted photons were recorded by the two-dimensional X-ray detector ePIX, while a beam block protected the detector from the primary beam. An ion time-of-flight detector positioned at the FEL focus recorded cluster fragments from the sample expansion, which occurs nanoseconds after the FEL exposure. Please see Methods for more information.

A detailed schematic of our experiment is shown in Fig. 1. Individual near-spherical Ne nanoparticles with diameters of 60 nm to 100 nm (0.5 × 10^7^ to 2.2 × 10^7^ atoms) intersect the path of intense single X-ray FEL pulses focused inside the LAMP end station at the Linac Coherent Light Source (LCLS)^46–48^. Single-exposure X-ray diffraction snapshots of nanoparticles are recorded at 60 Hz using the ePix detector^49^, which is located 0.395 m downstream from the interaction region. The particle size is encoded directly into Airy-pattern-like diffraction patterns and can be recovered with 0.3 nm accuracy^21,50^. Each diffraction image is correlated to a simultaneously recorded ion time-of-flight spectrum, which carries an imprint of the total energy absorbed by the nanoparticle^18^. Shorter flight times indicate higher ion energies. Both detectors and the Ne nanoparticle source are synchronized to the FEL pulse arrival times (see Methods).

We scan the photon energy *ℏω* in the vicinity of the Ne K-shell absorption edge between 815 eV and 1000 eV. Each scanning step contains thousands of diffraction patterns from individual Ne nanoparticles with fluctuating brightness due to random positions inside the 1.2 μm FEL focus with a near-Gaussian X-ray fluence distribution^18^ (see Methods).

**Fig. 2:**
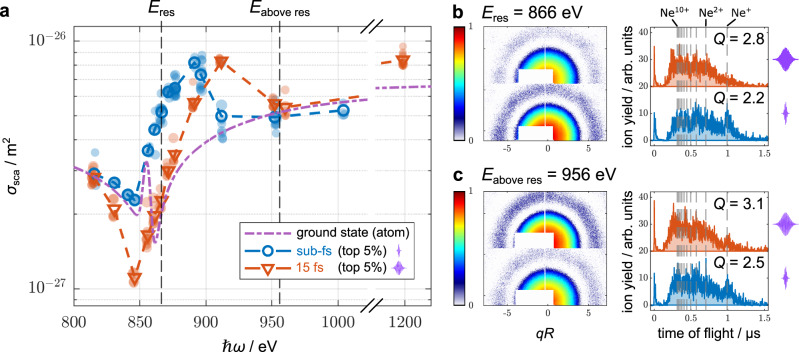
Diffraction and absorption trends in the vicinity of the Ne K-edge for sub-fs (blue) and 15-fs (red) exposures. Panel (**a**) exhibits the measured scattering cross-sections *σ*_sca_ of the brightest 5% of all normalized images for each photon energy *ℏω* (x-axis). Each dot represents a single diffraction pattern from a single nanoparticle. The simulated neutral state (purple dashed line) is displayed for comparison, see Suppl. Note 1 for details. The pre-edge transitions lead to a small maximum before the edge, which is broadened due to a convolution with a 6 eV Gaussian profile (FWHM) to account for the bandwidth of the FEL. The measured scattering cross-sections are amplified beyond the neutral state for certain energies for both pulse durations. However, the absorption increases less for sub-fs pulses. In panels (**b**, **c**), the average of the eight brightest diffraction patterns is displayed on the left side for photon energies 866 eV and 956 eV. On the right side, the corresponding average of the simultaneously recorded eight ion time-of-flight spectra are shown, respectively for both energies and pulse lengths. The absorbed energy can be measured through ion counts (y-axis) per ion time-of-flight (x-axis). The shorter the average time-of-flight, the more energy was absorbed. We converted the average time-of-flight into the corresponding average charge state *Q* (by neglecting the kinetic energy of ions). Ne charge states are the dotted vertical lines inside the ion spectra. Near the Ne K-edge resonance at 866 eV, the diffraction patterns for sub-fs pulses are brighter, but the corresponding average charge state is smaller compared to the 15 fs exposure (**b**). Above the resonance, this ratio is reversed. The brightest average image is connected to a higher average charge state. The diffraction patterns are plotted over the product between the scattering vector *q* and the nanoparticle radius *R*.

The measured variation of the absorbed energy and the diffraction cross-sections across the photon energy scan is exhibited in Fig. 2. From each individual diffraction pattern, we extracted the forward scattering intensity per Ne atom inside the nanoparticle (see Methods). The average atomic scattering cross-section can then be extracted by normalizing the scattering intensity to the incident FEL pulse fluence and, similar to previous studies, by assuming that the brightest of the normalized scattering intensities were produced near the FEL focus center^18,32,39^. The FEL pulse fluence is deduced from the pulse energy recorded shot-to-shot by an inline gas monitor detector. For details on the calculation and on the fitting routine for the nanoparticle size see Methods and Suppl. Notes 3 and 4a. In Fig. 2a, the scattering cross-sections *σ*_sca_ extracted from the top 5% of the brightest normalized patterns are plotted against the incoming photon energy *ℏω*. Each dot corresponds to a single image from an individual Ne nanoparticle. The observed cross-sections align overall with literature values for neutral Ne atoms (purple dashed line) except near the Ne K-shell resonance at 870 eV. Here, *σ*_sca_ is amplified by a factor of 2 to 3 compared to the neutral Ne curve for both pulse durations. The highest increase is close to 900 eV. Such behavior was already attributed to transient resonances in a previous study in Xe nanoparticles^39^. The intense FEL pulses create new ionic states, which can become resonant to the incoming X-ray photon energy. In the Xe nanoparticle study, the enhanced X-ray diffraction came at the cost of increased absorption efficiency for all pulse durations.

Our experimental data paints a more complex picture of the absorption vs. diffraction trends as displayed in panels b and c of Fig. 2. In panel b and c, an average of eight of the brightest snapshots are depicted per *τ* at two selected photon energies. The corresponding X-ray diffraction patterns (left) and their respective average ion spectra (right) recorded at photon energy ${E}_{{{{\rm{res}}}}}=866\,$ eV with *τ* ≈ 15 fs (b, top) and *τ* ≈ 0.3 fs pulses (b, bottom) are displayed. The diffraction image from the sub-fs pulse is brighter than that from the longer exposure. At the same time, the nanoparticles have absorbed less energy from the shortest pulse, as witnessed by a lower average charge state *Q* (b, right). This unusual behavior seems to disappear further above the K-edge at *E*_above res_ = 956 eV (see the panels in c). Here, the shorter exposure is also accompanied by a lower *Q* compared to the 15 fs exposure. In parallel, the X-ray diffraction recorded by the sub-fs pulses is dimmer, which suggests that increased scattering signal is linked to enhanced absorption similarly to previous observations^39^. The 15 fs pulses appear to generate brighter images at 956 eV. This corresponds to higher charge state generation, which has binding energies tens of eVs above the edge. Please note that *Q* does not reflect the exact average charge state during the exposure but is rather a relative measure of the total absorbed energy per atom. The charge state distribution detection occurs hundreds of nanoseconds after the FEL exposure. During the sample expansion significant cooling of the plasma occurs, which leads to a variety of recombination processes lowering the overall charge states. However, previous studies indicate that the expansion velocity and the resulting recombination processes are directly correlated to the total absorbed energy during the exposure^18,19,51^.

**Fig. 3:**
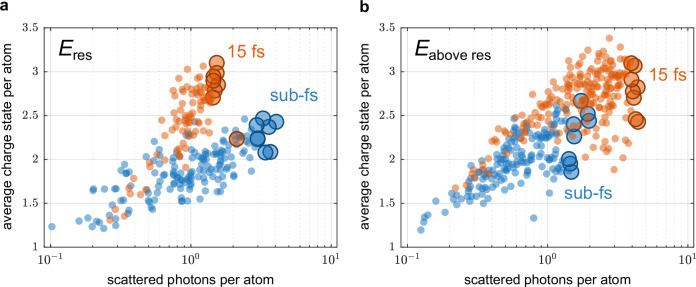
Statistical analysis of absorption vs scattering. A statistical analysis for all shots recorded at *E*_res_ (**a**) and *E*_above res_ (**b**) is shown. Each dot represents a single diffraction pattern and a corresponding ion spectrum from a single nanoparticle. The bold dots represent the eight brightest shots depicted in Fig. 2 on the right side for both aforementioned photon energies. In both panels, the photons scattered per Ne atom inside the individual nanoparticle (x-axis) are plotted versus the average charge state per atom *Q* (y-axis). At resonance depicted by the panel (**a**), the average charge state increases fast while the diffraction remains moderate for 15 fs exposures. In contrast, the images recorded with sub-fs exposure demonstrate a higher number of photons scattered per atom while the average charge state increases much slower. The slopes of correlations converge above the resonance in panel (**b**). Please note that the normalized number of photons per atom refers to forward scattering from isolated atoms. The normalization includes the impact of the measured size of the particles as described in Suppl. Note 4.

The selected images displayed in Fig. 2 represent a trend that emerges from the entire data set shown in Fig. 3. Here, the correlation between the number of scattered photons per Ne atom inside the nanoparticle (x-axis) is plotted versus the average charge states per ion *Q* extracted from the coincident ion spectra (y-axis). Each dot is a single FEL exposure with notable diffraction signal from a single nanoparticle. We calculate the number of photons scattered per atom from the radial profile fits of the scattering patterns and the Ne solid state density (see Suppl. Note 3). The simultaneously recorded ion time-of-flight spectra mirror the fraction of the FEL pulse energy absorbed by the nanoparticle. More absorbed X-ray photons result in a higher average charge state *Q* detected nanoseconds after the X-ray pulse. The larger dots are the eight brightest shots shown in Fig. 2. Panel a of Fig. 3 depicts the data recorded at ${E}_{{{{\rm{res}}}}}=866\,$ eV and panel b at *E*_above res_ = 956 eV, both recorded with sub-fs and 15 fs pulse durations. The FEL fluence distribution inside the FEL focus causes a wide spread of absorbed and diffracted X-ray energies. The 60 nm to 100 nm small particles are randomly injected into the much larger focal spot of the FEL with a full width at half maximum of  ~ 1.2 *μ*m. Thus, nanoparticles experience a broad variation of FEL fluences depending on their position inside the focal volume.

The overall trend is similar for both pulse durations; the number of scattered photons per atom (x-axis) increases with the average charge state (y-axis). However, the gradients of this trend diverge for sub-fs and 15 fs pulses at ${E}_{{{{\rm{res}}}}}$ and converge again at *E*_above res_. In panel a recorded at ${E}_{{{{\rm{res}}}}}$, the average charge state increases for 15-fs exposures without a significant enhancement of the scattering signal. In contrast, sub-fs exposures recorded near the FEL focus center witness more scattered photons per atom, whereas *Q* remains relatively moderate even in the brightest shots. In panel b, which exhibits data recorded at *E*_above res_, the increases in the average charge state and the scattering are very similar for both X-ray pulse durations.

Near the Ne K-edge, we observe in Fig. 2 and Fig. 3 that sub-fs FEL pulses are absorbed less efficiently but simultaneously produce stronger diffraction than few-femtosecond FEL pulses with a comparable fluence. This diverging behavior is unexpected in light of previous experimental studies. Earlier work in the XUV and soft X-ray regimes demonstrated enhanced absorption due to transient resonances^17,19,32,35,36,39,52^. These experiments, supported by simulations, showed that transient resonances increase absorption and thereby accelerate electronic excitation and ionic damage in the sample for all pulse durations. In contrast, the present results indicate a qualitatively different regime. Compared to the first reported sub-fs soft X-ray diffraction experiments^39^, the sub-fs FEL pulses used here deliver up to an order of magnitude higher pulse energy at the sample. This substantial increase in fluence is critical for accessing nonlinear light-matter interaction regimes, where processes such as stimulated emission can modify the net absorption throughout the pulse.

### Monte-Carlo/Molecular-Dynamics simulations of dynamic scattering cross-section

**Fig. 4:**
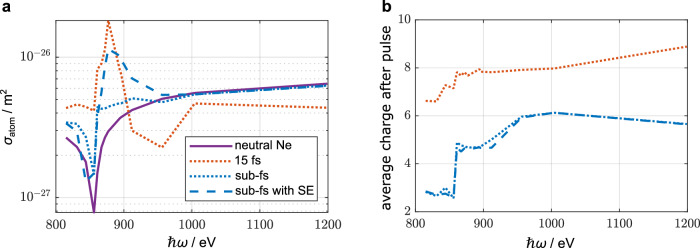
Simulation results. **a** We calculated the expected scattering cross-section per atom *σ*_atom_ inside the Ne nanoparticle exposed to FEL pulse conditions similar to the experimental focus center. The electronic damage after the FEL pulse is mirrored by the average charge state inside the nanoparticle right after the FEL pulse, as exhibited in (**b**). The calculations are plotted according to the incoming FEL photon energy *ℏω*. Dashed lines are simulations with stimulated emission, dotted lines without. Blue lines represent sub-fs exposures, red lines show 15 fs exposures. Similar to our experimental observation, the average charge state difference between both pulse durations at 866 eV and 956 eV are similar (around two). For further interpretation, see text and Suppl. Note 1.

We used our Monte Carlo-based calculation of nanoplasma effects inside Ne nanoparticles to model our experimental observations^25,32,39,53,54^ (see Methods). The simulation results are displayed in Fig. 4. Our calculations indicate that sub-femtosecond pulses with energies tuned to transient resonances can drive previously excited electrons back into the K-shell through stimulated emission. Thus, part of the absorbed energy from the X-ray pulse is re-emitted back into the light field. This de-excitation mechanism can become relevant if the pulses are shorter than Auger-Meitner decays and nanoplasma formation, which irreversibly dissipate the energy absorbed through photoionization further into the sample. Stimulated emission has two effects during the FEL exposure. First, some X-ray excitation inside the nanoparticle will be reversed by the release of stimulated emission through the incoming FEL. Second, the bound electrons remain longer resonant to the X-rays and scatter more efficiently throughout the exposure.

To test the hypothesis, we compare the elastic scattering cross-sections and the average charge states of Ne nanoparticles, which are exposed to FEL pulses close to the experimental conditions. In Fig. 4a, the calculated scattering cross-sections are plotted versus the incoming photon energy. Without stimulated emission, the scattering cross-sections produced by 300 attosecond FEL pulses (dotted blue line) are slightly above the neutral Ne case (solid purple line). If Ne nanoparticles are illuminated by 15 fs pulses, the scattering efficiency is elevated compared to the sub-fs exposure in the vicinity of the absorption edge and decreases for higher energies. This observation is reasonable, as there is a high density of transient resonances from Ne^1+^ to Ne^5+^ states, which can enhance the scattering efficiency prior to ionic disintegration of the sample (see Suppl. Note 1). Far above the edge, there are much fewer resonances and the amplification is suppressed. However, in the experiment we observe that sub-fs pulse exposures are scattered more efficiently in the vicinity of the K-edge than suggested by our first model.

When stimulated emission is included in the simulations (blue dashed line), the scattering cross-section rapidly surpasses the enhancements obtained with longer pulses at the same photon energy. This arises from repeated population transfer between resonantly excited and ionic ground states during the exposure. By maintaining a significant population in resonant configurations, stimulated emission prolongs their effective lifetime and amplifies diffraction over multiple scattering events.

Notably, this resonant enhancement occurs while the overall ionization level remains comparatively low. As shown in Fig. 4b (blue dotted line), the average charge state after sub-fs FEL irradiation lies between Ne^2+^ and Ne^4+^, corresponding to the absorption of up to three-four photons per atom. In contrast, 15 fs pulses produce substantially higher charge states, typically between Ne^6+^ and Ne^8+^ (red dotted line), driven mainly by Auger relaxation cascades and nanoplasma formation. According to our simulation, collisional ionization produces more than 50% of the total charge state for longer pulses. The number of collisional ionization events per atom is 4.4 for 815 eV and 4.9 at 877 eV for a 15 fs pulse. In this regime, a significant fraction of electrons contributing to X-ray scattering become delocalized from their parent ions. Although confined by the rapidly developing space-charge potential, they are no longer directly correlated to the positions of the original atoms.

For sub-fs pulses, however, the enhanced resonant scattering predominantly originates from electrons that remain bound to their ion. The resulting coherent diffraction patterns therefore closely reflect the pristine ionic arrangement, as atomic motion is effectively frozen on sub-femtosecond timescales. Our simulations further indicate that stimulated emission under these conditions overcomes electronic bleaching and continues to increase the scattering cross-section even when the X-ray fluence is raised by an order of magnitude (see Suppl. Fig. 2).

## Discussion

Overall, our study demonstrates that intense sub-fs FEL pulses can increase elastic X-ray diffraction efficiency in combination with decreased energy absorption in complex and dense nanoscale systems. Our simulation explains this behavior through two effects. First, sub-fs pulses outrun the Auger decay processes and most of the nanoplasma formation effects. Thus, X-ray diffraction occurs mainly on bound electrons. Second, stimulated emission from transient resonances works in favor of diffraction enhancement. Excited electrons can return to resonant states multiple times during the FEL exposure, and thus the scattering amplification becomes more likely. Interestingly, the overall charge state after the pulse is not greatly affected by stimulated emission as shown in Fig. 4b, dashed (stimulated emission included) vs dotted blue lines (no stimulated emission). On average, only a small portion of all ions emit stimulated emission, mostly at the peak of the pulse. This small fraction of ions suffices to increase the scattering cross-sections significantly, because the scattering cross-section of a single transient ion can be several orders of magnitude higher compared to the neutral Ne (see Suppl. Fig. 1). The highest transient resonance for Ne^9+^ is at 1600 eV and would in theory be able to provide sub-nm resolution images. These findings highlight the potential of ultrafast X-ray imaging with sub-nanometer resolution for light elements, which has been unlocked through the development of intense sub-fs X-ray FEL pulses. For heavier elements, sub-fs pulses in the hard X-ray regime can deliver similar effects at even shorter wavelengths.

In summary, our study establishes a new route for manipulating the X-ray refractive index in dense and complex nanosystems using intense sub-femtosecond FEL pulses. These ultrashort pulses, combined with extreme peak power, can outrun and even partially reverse electronic excitation through stimulated emission. This finding challenges the prevailing view that electronic bleaching is irreversible in experiments with intense X-ray pulses. In principle, the electronic state and X-ray induced damage can be coherently controlled via Rabi oscillations, enabled by the high temporal coherence of sub-fs FEL pulses. Rabi oscillations in isolated Ne^+^ ions have been suggested in the soft X-ray regime^35,44^ and observed experimentally in the XUV regime in He atoms^55,56^. Extending this concept from dilute atomic gases to dense, highly excited nanoplasmas opens a pathway toward coherent control of the X-ray refractive index in structurally complex systems.

In a macroscopic description based on Maxwell’s equations, stimulated emission can be regarded as akin to negative absorption, which is represented by the imaginary part of the refractive index. Thus, one can assume that stimulated emission will modify the scattered field beyond the forward direction. In the present experiment, this contribution mostly affects the brightness of the image. However, when stimulated emission occurs at higher rates, the angular distribution of scattered intensity might change, analogous to the role of absorption and dispersion in Mie scattering. Such effects would provide a direct experimental signature of stimulated emission in X-ray diffraction and offer new opportunities for enhancing material contrast.

More broadly, these results are expected to extend to other low-Z elements such as oxygen and nitrogen, which are central to organic and biological systems. In combination with hard X-rays, sub-femtosecond pulse durations may enable increased contrast and improved spatial resolution in diffraction-before-destruction experiments. By enabling partial suppression and coherent control of electronic excitation during exposure, our work represents a significant step toward direct visualization of chemical dynamics and ultrafast phase transitions with unprecedented spatial and temporal precision.

## Methods

### Experimental details

The experiment was performed at the time-resolved atomic, molecular and optical science (TMO) instrument^47,48^ of the linac coherent light source (LCLS)^46^. X-ray pulses were produced in two operation modes, by (a) the 'conventional’ self-amplified spontaneous emission (SASE) scheme with electron bunch pulse lengths of 15 fs to 20 fs, and (b) using the novel X-ray laser enhanced attosecond pulse generation (XLEAP) technique^40^, delivering near-Fourier limited sub-femtosecond pulses^40^. The actual X-ray pulse length is around two/thirds of the electron bunch, on average smaller than 15 fs. Table 1 shows an overview of the X-ray pulse parameters.

**Table 1:** The two pulse types used throughout the present experiment

pulse type	*τ*_FWHM_	〈*E*_p_〉 ± *σ*_p_	〈*ΔE*〉 ± *σ*_*ΔE*_
XLEAP	~ 0.3 fs	(62 ± 26) *μ*J	(6.4 ± 0.7) eV
SASE	10–15 fs	(114 ± 20) *μ*J	(5.6 ± 0.9) eV

The pulse duration (FWHM), pulse energy (mean  ± standard deviation) and energy bandwidth (mean ± standard deviation of FWHM at different energies) are denoted as *τ*_FWHM_, 〈*E*_p_〉 ± *σ*_p_ and 〈*ΔE*〉 ± *σ*_*ΔE*_, respectively. The pulse energies were recorded using a gas monitor detector (GMD) and corrected for beamline transmission.

A pair of Kirkpatrick–Baez mirrors focused the X-ray beam to  ~ 1.2 μm in diameter (full width at half maximum, FWHM) in the interaction point (IP). Fig. 1 shows a sketch of the experimental layout.

Clusters were produced by expansion of pre-cooled Ne gas into a vacuum chamber. The source employed a cryogenic Even–Lavie valve^57^ with a narrow conical nozzle ($\varnothing 100\,\upmu {{{\rm{m}}}}$, 2^∘^ half-opening angle) and operated at 60 Hz and 60 μs opening time. The stagnation pressure was set to 2 MPa at stagnation temperatures of 43 K to 47 K. For details on the cluster source setup please see refs. ^58,59^. The neon cluster beam passed through a 1 mm skimmer and a second piezo-driven variable-gap skimmer, allowing for hit rate reduction and single-shot single-particle operation.

X-rays diffracted by individual neon clusters were recorded with an ePix100 detector^60^, containing 768 × 704 pixels (50 × 50 μm^2^ pixel size) located 0.395 m after the IP. The X-ray photon detector was mounted shifted to one side of the beam stop, in order to capture the lowest-order diffraction. The detector covered diffraction angles up to 4.9^∘^ (edge), the Si beam stop blocked signal from angles below 0.7^∘^ and the direct FEL beam. An ion time-of-flight (iTOF) spectrometer with a 1 mm entrance slit was installed above the IP, recording coincident ion spectra. The slit prevents ions from outside of the Rayleigh length. See ref. ^23^ for details on the iTOF geometry and transmission function.

The ePix100 detector was movable and was driven completely out of the beam in order to complete the absolute photon energy calibration. A Fresnel zone plate (FZP) spectrometer (range: 821 eV to 886 eV), located behind the diffraction detector, was used for FEL photon energy calibration, see ref. ^61^ for details. FEL spectra were recorded for photon energies between 831 eV and 877 eV for XLEAP pulses, and between 844 eV and 872 eV for SASE pulses, respectively (calibrated values). A linear fit was used to calculate calibrated photon energies shot-to-shot from the nominal machine parameters. The FZP spectrometer was calibrated on the Ni L2 (870.0 eV) and L3 (852.7 eV) edges by tuning the FEL to the respective energy and recording spectra without and with a thin Ni absorption foil, for SASE and XLEAP operation, respectively.

### Processing of diffraction images

In the first step, the diffraction detector images were masked for inactive, dead, and hot pixels. In addition, pedestal, background, and common mode corrections were applied. Each experimental run contained approximately 18,000 FEL shots, from which the brightest 500 were selected for further analysis.

The nanoparticle size was extracted from the diffraction image as described below. Each detector pixel photon count ${N}_{{{{\rm{pix}}}}}^{{{{\rm{ph}}}}}$ corresponds to the time *t* integrated product of differential scattering cross-section d*σ*/d*Ω*, incident fluence ${{{\mathscr{F}}}}$, solid angle *ΔΩ*_pix_ of the pixel and quantum efficiency *D*_QE_

1 $${N}_{{{{\rm{pix}}}}}^{{{{\rm{ph}}}}}={{{\mathscr{F}}}}\Delta {\Omega }_{{{{\rm{pix}}}}}{D}_{{{{\rm{QE}}}}}\frac{{{{\rm{d}}}}\sigma }{{{{\rm{d}}}}\Omega }$$

The time-integrated scattered radiant intensity is then fitted to the scattering cross-section of a homogeneous sphere^50^:

2 $${{{\mathscr{J}}}}(q)=\frac{{N}_{{{{\rm{pix}}}}}^{{{{\rm{ph}}}}}(q)}{\Delta {\Omega }_{{{{\rm{pix}}}}}{D}_{{{{\rm{QE}}}}}}={{{\mathscr{F}}}}\frac{{{{\rm{d}}}}{\sigma }_{{{{\rm{sph}}}}}}{{{{\rm{d}}}}\Omega }\left(q\right)={{{{\mathscr{J}}}}}_{{{{\rm{sph}}}}}^{0}{\left[3\frac{\sin qR-qR\cos qR}{{q}^{3}{R}^{3}}\right]}^{2},$$

yielding the cluster radius *R* and integrated scattered radiant intensity into forward direction ${{{{\mathscr{J}}}}}_{{{{\rm{sph}}}}}^{0}$ as fit parameters. The magnitude of the elastic momentum transfer vector is given by $q=2k\,\sin (\theta /2)$, with the angular wave number *k* = 2*π*/*λ* and the diffraction angle *θ*.

The atomic scattering cross-section averaged over the entire XFEL pulse is given by

3 $${\sigma }_{{{{\rm{sca}}}}}=\frac{8\pi }{3}\frac{{{{{\mathscr{J}}}}}^{0}}{{{{\mathscr{F}}}}}\frac{1}{{N}_{{{{\rm{a}}}}}^{2}}.$$

*N*_a_ = 4*πR*^3^*n*_a_/3 is the number of atoms in the cluster, where *n*_a_ = 43 nm^−3^ is the number density of neon at the triple point^62^. The cluster radius *R* and the radiant intensity scattered into forward direction ${{{{\mathscr{J}}}}}^{0}$ were extracted from diffraction profile fits of near-spherical Ne nanoparticles. The incident fluence was calculated with ${{{\mathscr{F}}}}=\frac{2{E}_{{{{\rm{p}}}}}}{\pi {w}_{0}^{2}}$ from the total pulse energy *E*_p_, assuming a Gaussian beam profile with a beam waist of ${w}_{0}=\frac{{d}_{{{{\rm{FWHM}}}}}}{\sqrt{2\ln 2}}$ and beam diameter *d*_FWHM_ = 1.2 μm. Since only a minor fraction of the clusters were hit by the central beam, while most clusters were illuminated by the beam wings, the calculated scattering cross-section is underestimated in most cases. Therefore, only the top 5% of the calculated values for *σ*_sca_ are used to estimate the average atomic scattering cross-section for each pulse type, respectively. For further details, please refer to Suppl. Notes 3 and 4a.

### Monte-Carlo/Molecular-Dynamics simulations

We employed Monte-Carlo/Molecular-Dynamics (MC/MD) calculations to simulate the scattering cross-sections of the Ne clusters^32,39,53^ to model the full electron and nuclear dynamics in an atomistic manner during the full duration of the X-ray pulse. In more detail, the interaction of the atom with incident XFEL pulse is treated quantum mechanically with a Monte Carlo method by tracking explicitly the time-dependent quantum transition probability between different electronic configurations. The total transition rate *Γ* between different electronic configurations *I* and *J* is given by

4 $${\Gamma }_{I,J}={\Gamma }_{I,J}^{P}+{\Gamma }_{I,J}^{A}+{\Gamma }_{I,J}^{F}+{\Gamma }_{I,J}^{RE}+{\Gamma }_{I,J}^{EI}+{\Gamma }_{I,J}^{RC}+{\Gamma }_{I,J}^{SE}.$$

Starting from the ground state of the neutral atom, we include the contribution from photoionization ${\Gamma }_{I,J}^{P}$, Auger decay ${\Gamma }_{I,J}^{A}$, fluorescence ${\Gamma }_{I,J}^{F}$, resonant excitation ${\Gamma }_{I,J}^{RE}$, electron-impact ionization ${\Gamma }_{I,J}^{EI}$, electron-ion recombination ${\Gamma }_{I,J}^{RC}$ and stimulated emission rate ${\Gamma }_{I,J}^{SE}$. The cross-sections and rates are calculated with the Hartree-Fock-Slater model^25^ with relativistic corrections and spin-orbit coupling in orbital energies. The stimulated emission rates are derived from Einstein coefficients. Additionally, a molecular dynamics (MD) algorithm is used to propagate all particle trajectories (atoms/ions/electrons). The cluster dynamics includes electromagnetic forces between the charged particles and van der Waals forces among the neutral atoms.

The scattering response is characterized as a sum of the instantaneous scattering patterns weighted by the pulse intensity, *j*_*X*_(*τ*, *t*), with FWHM duration *τ* and convolved with a Gaussian bandwidth profile, *g*(*ω*, *ω*_*x*_), with a central photon energy of *ω*_*x*_, such that

5 $$\frac{{{{\rm{d}}}}\sigma }{{{{\rm{d}}}}\Omega }=\frac{{{{\rm{d}}}}{\sigma }_{{{{\rm{th}}}}}}{{{{\rm{d}}}}\Omega }\frac{1}{{{{\mathscr{F}}}}}\int_{0}^{+\infty }\,\,{{{\rm{d}}}}\omega \int_{-\infty }^{+\infty }\,\,{{{\rm{d}}}}t\,g(\omega,{\omega }_{x})\,{j}_{X}(\tau,t)| {F}_{c}(\vec{q},t){| }^{2},$$

where d*σ*_th_/d*Ω* is the Thomson scattering cross-section.

6 $${{{\mathscr{F}}}}=\int_{0}^{+\infty }\,\,{{{\rm{d}}}}\omega \int_{-\infty }^{+\infty }\,\,{{{\rm{d}}}}t\,{j}_{X}(\tau,t)g(\omega,{\omega }_{x})$$

is the fluence of an XFEL pulse, and $\int_{0}^{+\infty }\,\,{{{\rm{d}}}}\omega \,g(\omega,{\omega }_{x})=1$. Here ${F}_{c}(\vec{q},t)$ is the time-dependent form factor of the target cluster and is modeled as the sum of the form factors of all ions/atoms (${F}_{a}(\vec{q},t)$) and electrons (${F}_{e}(\vec{q},t)$). Here,

7 $${F}_{a}(\vec{q},t)={\sum}_{j=1}^{{N}_{a}}{f}_{j}(\vec{q},{C}_{j}(t)){e}^{i\vec{q}\cdot {\vec{R}}_{j}(t)}\,,$$

where *N*_*a*_ is the total number of atoms/ions, ${\vec{R}}_{j}(t)$, *C*_*j*_(*t*) and ${f}_{j}(\vec{q},{C}_{j}(t))$ are the position, the electronic configuration and the atomic form factor of the *j*-th atom/ion respectively. To capture the effect of delocalized electrons in a large cluster, the electrons are assumed to distribute uniformly within the cluster with size *R*, such that

8 $${F}_{e}(\vec{q},t)=\frac{3{N}_{e}(t)(\sin (qR)-qR\cos (qR))}{{(qR)}^{3}}\,,$$

where *N*_*e*_(*t*) is the number of delocalized electrons within the focal region of the X-ray pulse. A more detailed discussion of the simulations can be found in Supplementary Note 1.

## Supplementary information

Supplementary Information

Transparent Peer Review file

## Data Availability

The fit results data of the 500 brightest detector images per experimental run have been deposited in the Zenodo database under accession code 20633871. Additionally, the raw area detector images and time-of-flight spectra were uploaded for the brightest 100 shots per experimental run under accession code 20633891.
