# Supplementary material for: Nonlinear reversal of photoexcitation on the attosecond time scale improves ultrafast X-ray diffraction images
Source: Nat Commun. 2026 Jul 28;17:7556. doi: 10.1038/s41467-026-75969-8 (PMC13415538; doi:10.1038/s41467-026-75969-8)
Supplement: Supplementary file 1 — Supplementary Information [file 41467_2026_75969_MOESM1_ESM.pdf]

Supplementary Information

Nonlinear reversal of photoexcitation on the attosecond time scale improves ultrafast X-ray diffraction images

SUPPLEMENTARY NOTE 1: MONTE-CARLO/MOLECULAR-DYNAMICS SIMULATIONS

We employed Monte-Carlo/Molecular-Dynamics (MC/MD) calculations to simulate the scattering cross-sections of the Ne clusters [1–3] to model the full electron and nuclear dynamics in an atomistic manner during the full duration of the X-ray pulse. In more detail, the interaction of the atom with incident XFEL pulse is treated quantum mechanically with a Monte Carlo method by tracking explicitly the time-dependent quantum transition probability between different electronic configurations. The total transition rate Γ between different electronic configurations I and J is given by

$$\Gamma_{I,J} = \Gamma^{P}_{I,J} + \Gamma^{A}_{I,J} + \Gamma^{F}_{I,J} + \Gamma^{RE}_{I,J} + \Gamma^{EI}_{I,J} + \Gamma^{RC}_{I,J} + \Gamma^{SE}_{I,J}. \quad (1)$$

Starting from the ground state of the neutral atom, we include the contribution from photoionization $\Gamma^{P}_{I,J}$, Auger decay $\Gamma^{A}_{I,J}$, fluorescence $\Gamma^{F}_{I,J}$, resonant excitation $\Gamma^{RE}_{I,J}$, electron-impact ionization $\Gamma^{EI}_{I,J}$, electron-ion recombination $\Gamma^{RC}_{I,J}$ and stimulated emission rate $\Gamma^{SE}_{I,J}$. The cross-sections and rates are calculated with the Hartree-Fock-Slater model [4] with relativistic corrections and spin-orbit coupling in orbital energies. The stimulated emission rates are derived from Einstein coefficients. Additionally, a molecular dynamics (MD) algorithm is used to propagate all particle trajectories (atoms/ions/electrons). The cluster dynamics includes electromagnetic forces between the charged particles and van der Waals forces among the neutral atoms.

The scattering response is characterized as a sum of the instantaneous scattering patterns weighted by the pulse intensity, $j_X(\tau, t)$, with FWHM duration τ and convolved with a Gaussian bandwidth profile, $g(\omega, \omega_x)$, with a central photon energy of ω_x, such that

$$\frac{\mathrm{d}\sigma}{\mathrm{d}\Omega} = \frac{\mathrm{d}\sigma_{\mathrm{th}}}{\mathrm{d}\Omega}\frac{1}{\mathscr{F}}\int_0^{+\infty}\mathrm{d}\omega\int_{-\infty}^{+\infty}\mathrm{d}t\, g(\omega, \omega_x) j_X(\tau, t)|F_c(\vec{q}, t)|^2, \quad (2)$$

where $\mathrm{d}\sigma_{\mathrm{th}}/\mathrm{d}\Omega$ is the Thomson scattering cross-section.

$$\mathscr{F} = \int_0^{+\infty} \mathrm{d}\omega \int_{-\infty}^{+\infty} \mathrm{d}t \, j_X(\tau, t) g(\omega, \omega_x) \tag{3}$$

is the fluence of an XFEL pulse, and $\int_0^{+\infty} \mathrm{d}\omega \, g(\omega, \omega_x) = 1$. Here $F_c(\vec{q}, t)$ is the time-dependent form factor of the target cluster and is modeled as the sum of the form factors of all ions/atoms ($F_a(\vec{q}, t)$) and electrons ($F_e(\vec{q}, t)$). Here,

$$F_a(\vec{q}, t) = \sum_{j=1}^{N_a} f_j(\vec{q}, C_j(t)) e^{i\vec{q}\cdot\vec{R}_j(t)} , \tag{4}$$

where N_a is the total number of atoms/ions, $\vec{R}_j(t)$, $C_j(t)$ and $f_j(\vec{q}, C_j(t))$ are the position, the electronic configuration and the atomic form factor of the j-th atom/ion respectively. To capture the effect of delocalized electrons in a large cluster, the electrons are assumed to distribute uniformly within the cluster with size R, such that

$$F_e(\vec{q}, t) = \frac{3N_e(t)(\sin(qR) - qR\cos(qR))}{(qR)^3} , \tag{5}$$

where $N_e(t)$ is the number of delocalized electrons within the focal region of the X-ray pulse.

Our simulation demonstrated in Fig. 4 overall agrees with the experimental data shown in Fig. 3, especially with the set recorded with sub-fs pulses. Stimulated emission extends the overall lifetime of a small fraction of transient ions (less than 10-15 percent). However, this fraction suffices to increase the image brightness significantly.

Our simulation indicates that near the Ne K-edge, the average charge state inside the nanoparticle is around Ne^{4+} right after the FEL exposure. During the exposure, there is a charge state distribution ranging from 1+ to up to 5+. In Supplementary Fig. 1, the scattering cross-sections for different Ne ions are plotted versus incoming photon energy. In case (a), where a bandwidth smaller than 1 eV is assumed, the amplification of diffraction can go up to 6 orders of magnitude (!) as witnessed by the Ne^{7+} curve (dark red line) near 900 eV. If convoluted with the 6 eV bandwidth of sub-fs pulses, the increase in scattering can still go up by 4 orders of magnitude. Thus, even a small fraction of resonant ions throughout the exposure will increase the total scattering cross-section per ion inside the nanoparticle, especially if the lifetime of such resonances is increased through stimulated emission.

There are two discrepancies compared to the experiment, mostly in the plot tracing the 15 fs data set. First, the simulation indicates that there is an elevation of scattering cross-section below the Ne edge, which is not present in the data. This behavior might come

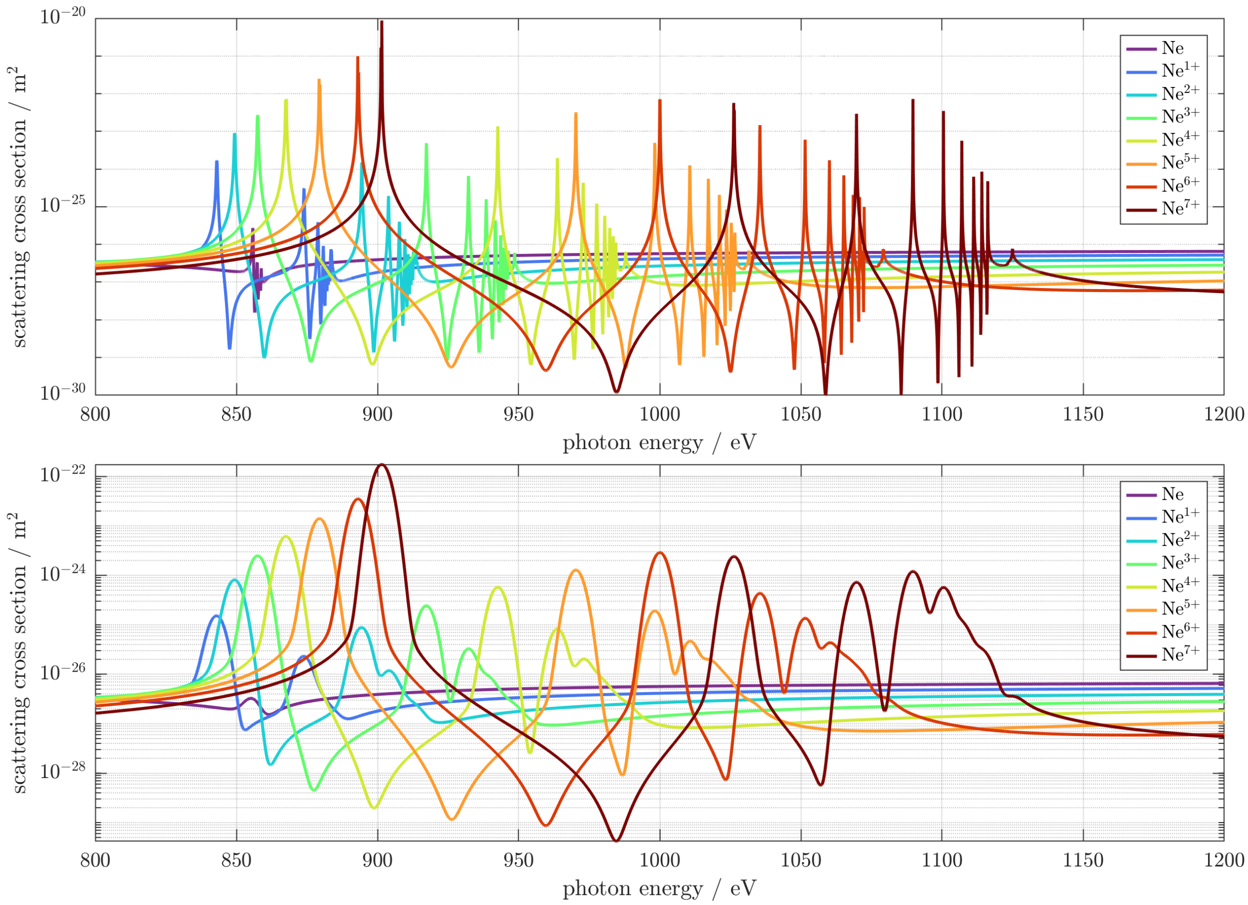

SUPPLEMENTARY FIG. 1. (a) Calculated scattering cross-section of different Ne charge states plotted vs incoming X-ray photon energy. (b) Same data as in (a) but convolved with a 6 eV-Gaussian profile (FWHM) to account for the spectral width of the FEL.

from a phase shift between the quasi-free electrons and the remaining bound electrons. The phase shift mentioned here does not enter as an explicit parameter in the present model. Rather, it would modify the relative phase between the bound-electron form factor $F_a(q,t)$ and the delocalized-electron contribution $F_e(q,t)$, thereby changing the interference term in $|F_c(q,t)|^2$. In the current implementation, quasi-free electrons are treated as uniformly distributed and phase-averaged, which may underestimate such interference effects in rapidly evolving nanoplasma potentials. Rapid charging during the pulse can dynamically reshape the cluster potential, potentially inducing additional phase shifts not fully captured in the present simulation. This evolving potential may also explain the second deviation: the slight mismatch between experiment and simulation of the photon energies with the highest scattering cross-sections.

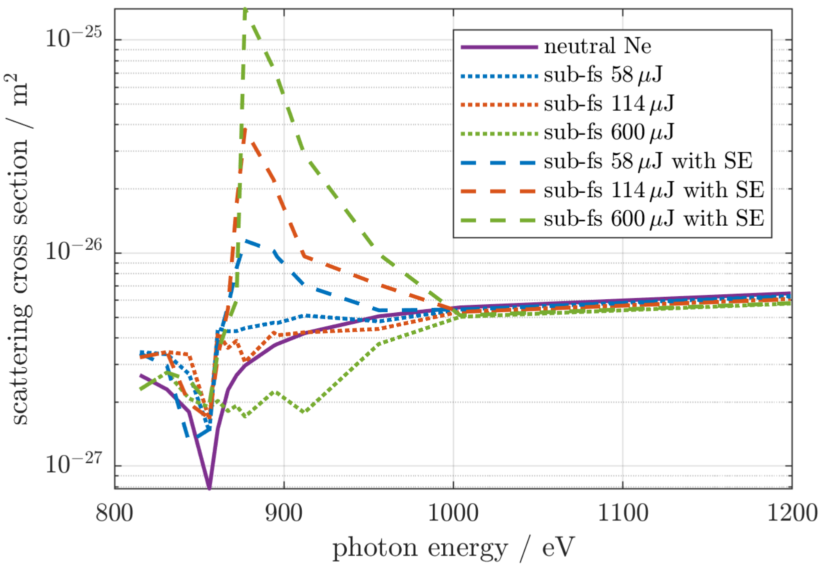

SUPPLEMENTARY FIG. 2. Simulated scattering cross-section for neutral Ne atoms inside the nanoparticles (purple solid line), sub-fs pulses with different FEL pulse energies without stimulated emission (dotted lines) and with simulation emission (dashed lines).

In Supplementary Fig. 1, the calculated Ne ion cross-sections curves up to Ne^{7+} are plotted vs the incoming pulse energy. The neutral case is the purple curve. The first maximum of each curve are 1s - 3p transitions, each additional maximum are transitions into higher n shells. We don't expect bound states beyond $n = 3$ to survive the potential suppression during nanoplasma formation, thus for our experiment most likely resonances up to 900 eV play the most crucial role.

SUPPLEMENTARY NOTE 2: SCALING OF IMAGE BRIGHTNESS WITH SUB-FS PULSES

Our simulation strongly suggests that stimulated emission will play a significant role in improving the brightness and ultimately the resolution of X-ray diffraction images. In Supplementary Fig. 2 we plot the results of a thought experiment, where we increase the FEL pulse energy stepwise from 0.05 mJ to 0.6 mJ. Here, the scattering cross-sections continue to rise around 900 eV with the pulse energy if the stimulated emission is included in the simulation. Compared to the neutral curve, one can gain almost two orders of magnitude in image brightness beyond scattering from neutral Ne (purple solid curve). Without stim-

ulated emission, we expect to see effects from electronic bleaching even for sub-fs pulses (see dotted green curve). Our thought experiment demonstrates that sub-fs pulses not only beat bleaching by outrunning the nanoplasma formation but that the diffraction signal from bound electrons becomes dominant when scaling through brightness of images through increase X-ray fluorescence. Bound electrons reflect the pristine structure of the ion positions inside the sample, and thus sub-fs pulses are ideal candidates for true diffraction-before-destruction imaging with atomic resolution.

SUPPLEMENTARY NOTE 3: EXTRACTING SCATTERING CROSS-SECTIONS FROM DIFFRACTION PATTERNS

The differential and total scattering cross-sections for an electron (Thomson cross-section) are respectively given by [5]:

$$\frac{\mathrm{d}\sigma_\mathrm{e}}{\mathrm{d}\Omega} = r_\mathrm{e}^2 \sin^2\Theta \qquad \text{and} \qquad \sigma_\mathrm{e} = \frac{8\pi}{3} r_\mathrm{e}^2 \tag{6}$$

where Θ is the angle measured from the axis of acceleration $\mathbf{a}$, accounting for polarization. The differential and total scattering cross-sections for an atom in the case of long wavelength ($\lambda \gg a_0$) or small angles ($\theta \ll \lambda/a_0$) are respectively given by [5]:

$$\frac{\mathrm{d}\sigma_\mathrm{a}}{\mathrm{d}\Omega} = r_\mathrm{e}^2 \left|f^0(\omega)\right|^2 \sin^2\Theta \quad \text{and} \quad \sigma_\mathrm{a} = \frac{8\pi}{3} r_\mathrm{e}^2 \left|f^0(\omega)\right|^2 \tag{7}$$

Here $f^0(\omega)$ denotes the neutral atomic forward scattering factor (atomic form factor in the limit of small momentum transfer), which describes the elastic scattering amplitude of a neutral neon atom. This quantity corresponds to the configuration-dependent atomic form factor $f_j(\mathbf{q}, C_j)$ introduced in the Theory section in the limit of a neutral atom and forward scattering ($\mathbf{q} \to 0$).

The angular distribution of X-rays scattered by a homogeneous sphere with Radius R is given by [6, 7]

$$\frac{\mathrm{d}\sigma_\mathrm{sph}}{\mathrm{d}\Omega} = \left(\frac{\mathrm{d}\sigma_\mathrm{a}}{\mathrm{d}\Omega}\right) 8\pi^3 R^6 n_\mathrm{a}^2 \left(\frac{J_{3/2}(qR)}{(qR)^{3/2}}\right)^2 \tag{8}$$

$$= \left(\frac{\mathrm{d}\sigma_\mathrm{a}}{\mathrm{d}\Omega}\right) N_\mathrm{a}^2 \left[3\frac{\sin qR - qR\cos qR}{q^3R^3}\right]^2 \tag{9}$$

where $q = 2k\sin(\theta/2)$, $k = 2\pi/\lambda$ is the angular wavenumber with wavelength λ, $N_\mathrm{a} = n_\mathrm{a}\, 4\pi R^3/3$ is the number of atoms, J is a Bessel function of the first kind and $(\mathrm{d}\sigma_\mathrm{a}/\mathrm{d}\Omega)$ is

the differential cross-section of a single atom. Here n_a denotes the atomic number density of neon, assumed to be uniform within the nanoparticle. The total scattered power can be calculated by integrating the radiant intensity over all solid angles $\mathrm{d}\Omega = \sin\theta\,\mathrm{d}\theta\mathrm{d}\phi$:

$$P_{\text{sca}} = \int I_{r,\Omega}\,\mathrm{d}\Omega = j^0_{\text{sca}} \int_0^{2\pi}\int_0^{\pi} \left[3\frac{\sin qR - qR\cos qR}{q^3R^3}\right]^2 \sin\theta\,\mathrm{d}\theta\mathrm{d}\phi = \frac{9\pi}{2k^2R^2} j^0_{\text{sca}} \tag{10}$$

On the other hand we have

$$\frac{\mathrm{d}\sigma_{\text{sph}}}{\mathrm{d}\Omega} = \frac{1}{|S_{\text{inc}}|}\frac{\mathrm{d}P_{\text{sca}}}{\mathrm{d}\Omega} = \frac{j_{\text{sca}}(\theta,\phi)}{I_{\text{inc}}} = \frac{j^0_{\text{sca}}}{I_{\text{inc}}}\left[3\frac{\sin qR - qR\cos qR}{q^3R^3}\right]^2 \tag{11}$$

$$\Rightarrow \sigma_{\text{sph}} = \int \frac{j_{\text{sca}}}{I_{\text{inc}}}\mathrm{d}\Omega = \frac{j^0_{\text{sca}}}{I_{\text{inc}}} \int_0^{2\pi}\int_0^{\pi} \left[3\frac{\sin qR - qR\cos qR}{q^3R^3}\right]^2 \sin\theta\,\mathrm{d}\theta\mathrm{d}\phi = \frac{9\pi}{2k^2R^2}\frac{j^0_{\text{sca}}}{I_{\text{inc}}} \tag{12}$$

Combining the two relations yields

$$\left(\frac{\mathrm{d}\sigma_{\text{a}}}{\mathrm{d}\Omega}\right) = \frac{j^0_{\text{sca}}}{I_{\text{inc}}}\frac{1}{N_{\text{a}}^2} = r_{\text{e}}^2\left|f^0(\omega)\right|^2\cos^2\Theta \xrightarrow{q\to 0} r_{\text{e}}^2\left|f^0(\omega)\right|^2 = \frac{j^0_{\text{sca}}}{I_{\text{inc}}}\frac{1}{N_{\text{a}}^2} \tag{13}$$

allowing to compare the scattering cross-section per atom

$$\sigma_{\text{a}} = \frac{8\pi}{3} r_{\text{e}}^2\left|f^0(\omega)\right|^2 = \frac{8\pi}{3}\frac{j^0_{\text{sca}}}{I_{\text{inc}}}\frac{1}{N_{\text{a}}^2} \tag{14}$$

with tabulated atomic scattering factor values if irradiance I_{inc} and scattered radiant intensity j^0_{sca} are known. For the present case, the time-integrated irradiance and scattered radiant intensity

$$\mathscr{F} = \int_{-\infty}^{+\infty} \mathrm{d}t\, I_{\text{inc}} \qquad \text{and} \qquad \mathscr{J} = \int_{-\infty}^{+\infty} \mathrm{d}t\, j_{\text{sca}} \tag{15}$$

are measured. Hereby $\mathscr{F}$ is just the fluence of the pulse in the FEL focus and $\mathscr{J}$ is proportional to the angle-dependent photon count on the 2D area detector. Therefore, the atomic scattering cross-section averaged over the entire XFEL pulse is given by

$$\sigma_{\text{sca}} = \frac{8\pi}{3}\frac{\mathscr{J}^0}{\mathscr{F}}\frac{1}{N_{\text{a}}^2}. \tag{16}$$

SUPPLEMENTARY NOTE 4: DATA PROCESSING

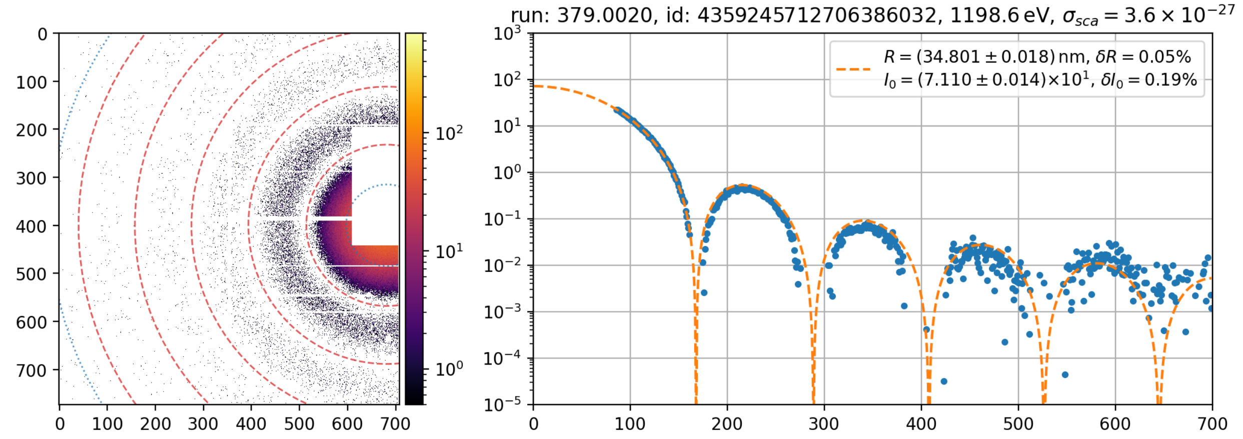

SUPPLEMENTARY FIG. 3. Example diffraction image from an individual nanoparticle (left; x- and y-axis are pixel numbers) and the corresponding one-dimensional radial profile (right; x-axis is radial pixel number, y-axis is average photon count). The diffraction signal (blue dots) is fitted by the shape of a phase-shifting sphere as described below. The fit is shown in as dashed line, the locations of fitted minima is indicated through dashed line in the 2D diffraction image.

Supplementary Note 4.a: Diffraction Images

In the first step, the diffraction detector images were masked for inactive, dead and hot pixels. In addition, pedestal, background, and common mode corrections were applied. Each experimental run contained approximately 18000 FEL shots, from which the brightest 500 were selected for further analysis.

The nanoparticle size was extracted from the diffraction image as shown in Supplementary Fig. 3 and described below. Each detector pixel photon count $N_{\mathrm{pix}}^{\mathrm{ph}}$ corresponds to the time t integrated product of differential scattering cross-section $\mathrm{d}\sigma/\mathrm{d}\Omega$, incident fluence $\mathscr{F}$, solid angle $\Delta\Omega_{\mathrm{pix}}$ of the pixel and quantum efficiency D_{QE}

$$N_{\mathrm{pix}}^{\mathrm{ph}} = \mathscr{F}\Delta\Omega_{\mathrm{pix}} D_{\mathrm{QE}} \frac{\mathrm{d}\sigma}{\mathrm{d}\Omega} \tag{17}$$

The time-integrated scattered radiant intensity is then fitted to the scattering cross-section of a homogeneous sphere [8]:

$$\mathscr{I}(q) = \frac{N_{\mathrm{pix}}^{\mathrm{ph}}(q)}{\Delta\Omega_{\mathrm{pix}} D_{\mathrm{QE}}} = \mathscr{F}\frac{\mathrm{d}\sigma_{\mathrm{sph}}}{\mathrm{d}\Omega}(q) = \mathscr{I}_{\mathrm{sph}}^{0}\left[3\frac{\sin qR - qR\cos qR}{q^3R^3}\right]^2, \tag{18}$$

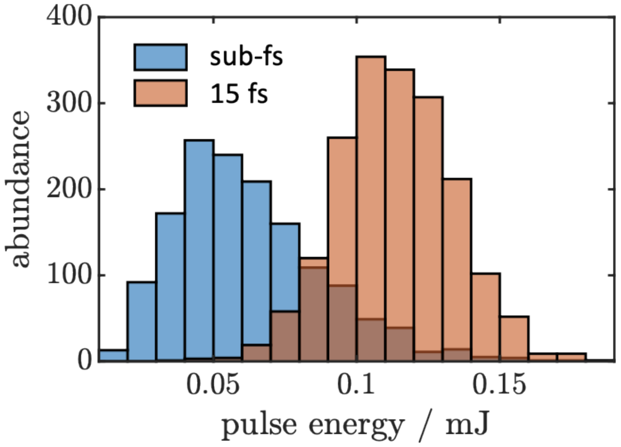

SUPPLEMENTARY FIG. 4. Pulse energy histograms (on target) for the analyzed data set. Only shots with good hits and near-spherical nanoparticles are shown. The distribution of sub-fs pulses has a tail towards higher pulse energies, which is not present in the Gaussian-like distribution of 15-fs pulses. In order to cover the most intense sub-fs pulses, the average pulse energy of 15-fs pulses was shifted to higher pulse energies

yielding the cluster radius R and integrated scattered radiant intensity into forward direction $\mathscr{I}^0_{\mathrm{sph}}$ as fit parameters. The magnitude of the elastic momentum transfer vector is given by $q = 2k\sin(\theta/2)$, with the angular wave number $k = 2\pi/\lambda$ and the diffraction angle θ. The wavelength-dependent quantum efficiency D_{QE} of the scattering detector increases approximately linearly from 11% to 44% for photon energies ranging from 800 eV to 1200 eV. The scattering cross-section per atom can be calculated using Supplementary Eq. (16). $N_{\mathrm{a}} = 4\pi R^3 n_{\mathrm{a}}/3$ is the number of atoms in the cluster, where $n_{\mathrm{a}} = 43\,\mathrm{nm}^{-3}$ is the number density of neon at the triple point [9]. The incident fluence was calculated with $\mathscr{F} = 2E_{\mathrm{p}}/\pi w_0^2$ from the total pulse energy E_{p}, assuming a Gaussian beam profile with a beam waist of $w_0 = d_{\mathrm{FWHM}}/\sqrt{2\ln 2}$ and beam diameter $d_{\mathrm{FWHM}} = 1.2\,\mu\mathrm{m}$ (see discussion further below). Histograms of the pulse energies of the evaluated data set are shown in Supplementary Fig. 4. All processed images were checked for the alignment of the fit to the data. Multiple particle hits or fits greatly diverging from a sphere were excluded. Additionally, only clusters with radii between 20 nm and 50 nm were selected for further analysis, to improve comparability and ensure that absorption effects are minute. Size distributions of the evaluated data set can be found in Supplementary Fig. 5.

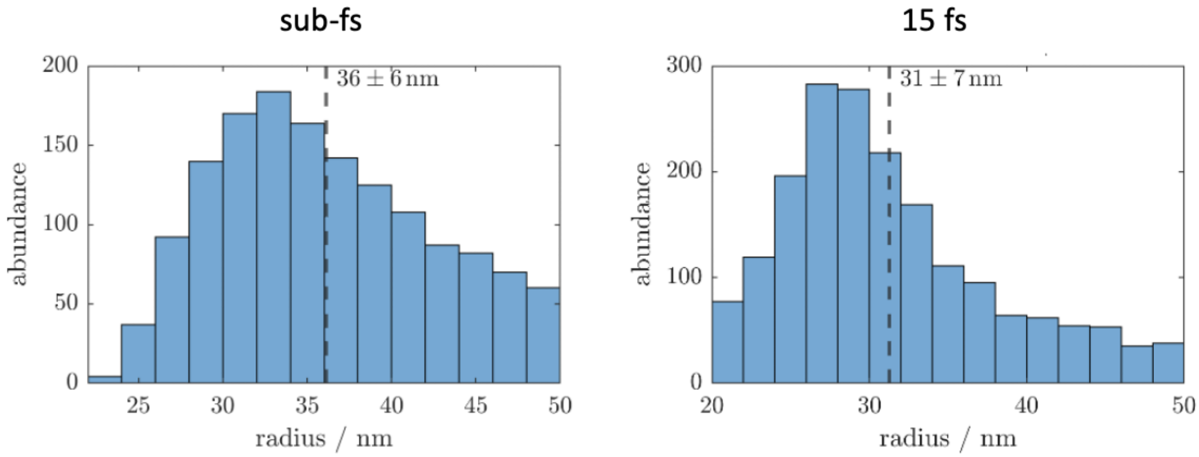

SUPPLEMENTARY FIG. 5. Radius histograms of the clusters (filtered for 20 nm to 50 nm) used throughout the present analysis. The different distributions are presumably resulting from differences in the detection efficiency due to different photon fluences of the individual pulse types. The (mean ± standard) deviation values for the two pulse types are (36 ± 6) nm for XLEAP and (31 ± 7) nm for SASE attenuated.

Since only a minor fraction of the clusters were hit by the central beam, while most clusters were illuminated by the beam wings, the calculated scattering cross-section is underestimated in most cases. Therefore, only the top 5% of the calculated values for σ_{sca} are used to estimate the average atomic scattering cross-section $\langle\sigma^j_{\text{sca}}\rangle_{5\%}$ for each pulse type $j \in \{\text{XLEAP, SASE}\}$.

$$\langle\sigma^j_{\text{sca}}\rangle_{5\%} = \text{mean}\left[\{\sigma^j_{\text{sca},i}\} \geq \eta_{95\%}(\{\sigma^j_{\text{sca},i}\})\right] \tag{19}$$

with $\eta_{95\%}(\{\sigma^j_{\text{sca},i}\})$ denoting the 95th percentile of the set of shots $\{\sigma^j_{\text{sca},i}\}$. The beam diameter is known to be $\gtrsim 1.2\,\mu\text{m}$ from wavefront measurements [10]. We confirmed the value based on linear regime scattering below the Ne absorption edge. For the clusters which were illuminated with sub-fs pulses below the edge (around 815 eV) approximately one photon per atom may be absorbed and no transient resonances are present. Thus, the diffraction should follow literature values. Fitting $\langle\sigma^{\text{X}}_{\text{sca}}\rangle_{5\%}(815\,\text{eV})$ to the literature value of the linear atomic scattering cross-section [6] yields a beam diameter of $d_{\text{FWHM}} = 1.17\,\mu\text{m}$, which is in good agreement with the expected value from the wavefront measurements.

Supplementary Note 4.b: Time-of-Flight Spectra

The ion time-of-flight (iTOF) spectra were recorded coincidently with the scattering patterns using a spectrometer described in detail in Ref. [11]. Due to an impedance mismatch at the digitizer, the signal was distorted by reflections. In a first step, the signal was corrected by subtracting a moving background (using the MATLAB [12] 'msbackadj' function with parameters: window size = 50 samples, step size = 25 samples, quantile = 0.05, regression method = 'pchip'). The flight times were calibrated using peaks of $^{20}Ne^{Q+}$ and $^{22}Ne^{Q+}$ with charges Q ranging from 2 to 8 from spectra of dilute neon gas. While iTOF spectra of neon gas show sharp peaks for the element specific m/Q values, the spectra of clusters are broadened by the kinetic energies the ions gain from interaction with the electric field of the other cluster fragments. In neon gas only ionization states up to Ne^{8+} is accessible by photon energies below 1200 eV [13]. Therefore, ions with shorter flight times indicate either additional ionization channels within the cluster, e.g. through electron impact ionization in the plasma; or (probably mainly) kinetic energy from the acceleration resulting from larger space charges. Both of these indicate increased absorption in the cluster, which is why the average cluster charge calculated from the average flight time is used as an indicator for absorption, even if it does not necessarily directly map to real ionization states. In addition, one should note, that ion spectra are signatures of nanoplasma, which had microseconds of recombination after the FEL has passed. In previous studies, ion spectra were reliably used to estimate the overall absorbed energy from the FEL pulses, but not for the measurement of charge states during the FEL exposure [14–16].

SUPPLEMENTARY NOTE 5: INFLUENCE OF SUB-FS FEL PULSE BANDWIDTH ON STIMULATED EMISSION

In our experiment, the measured FEL bandwidth is approximately 6 eV (see Table I), which is consistent with previous measurements of sub-fs FEL pulses reported by Duris et al. [17]. While this bandwidth is not negligible, its impact on stimulated emission depends critically on the structure and separation of the relevant resonances. There are two key aspects to consider. First, despite the finite bandwidth, the ionic resonances remain well resolved and pronounced. This is demonstrated in Supplementary Fig. 1(b), where the calculated response including the measured FEL bandwidth still shows clear resonant features.

This occurs because the relevant core–excited ionic transitions are separated in energy by amounts comparable to or larger than the FEL bandwidth.

Second, although broadband excitation can in principle enhance incoherent processes, particularly for neutral Ne where the 1s–3p transition lies close to the continuum, our simulations show that stimulated emission from neutral Ne is negligible under our experimental conditions. This result is physically expected because, at the highest FEL intensities used here, each Ne atom absorbs on average more than 2–5 photons per pulse, leading to rapid ionization and depletion of the neutral population. The dominant contribution to stimulated emission in our experiment instead arises from ionic species, primarily Ne^{2+}, Ne^{3+}, and Ne^{4+}, involving 1s–2p and 1s–3p transitions. In these ions, the excited states are well separated from the continuum and from neighboring excited levels. For example, in Ne^{2+} the 3p level lies approximately 25 eV below the continuum, while the next higher 4p level is separated by about 10 eV. Both energy separations exceed the FEL bandwidth. As a result, these transitions can be treated to a good approximation as isolated two-level systems, for which stimulated emission remains efficient even in the presence of finite spectral bandwidth.

We note that nanoplasma effects, such as ionization potential depression, can shift resonance energies and modify transition strengths. However, these shifts are expected to remain comparable to or smaller than the level spacings discussed above and therefore do not qualitatively change our conclusion that the relevant ionic resonances remain spectrally distinguishable within the FEL bandwidth. Furthermore, the ultrashort sub-fs pulse duration ensures that stimulated emission occurs on a timescale comparable to or faster than competing incoherent processes such as impact ionization and Auger decay, which become more significant only for longer (few-fs) pulses. In summary, although the FEL bandwidth is finite, it remains sufficiently narrow compared to the relevant ionic level separations to preserve the resonant character of the transitions responsible for stimulated emission.

REFERENCES

[1] P. J. Ho, C. Knight, M. Tegze, *et al.*, Atomistic three-dimensional coherent x-ray imaging of nonbiological systems, Phys. Rev. A **94**, 063823 (2016).

[2] P. J. Ho, B. J. Daurer, M. F. Hantke, *et al.*, The role of transient resonances for ultra-fast imaging of single sucrose nanoclusters, Nat. Commun. **11**, 167 (2020).

[3] S. Kuschel, P. J. Ho, A. Al Haddad, *et al.*, Non-linear enhancement of ultrafast X-ray diffraction through transient resonances, Nat. Commun. **16**, 847 (2025).

[4] P. J. Ho and C. Knight, Large-scale atomistic calculations of clusters in intense x-ray pulses, J. Phys. B **50**, 104003 (2017).

[5] D. Attwood and A. Sakdinawat, *X-Rays and Extreme Ultraviolet Radiation* (Cambridge University Press, 2016).

[6] B. L. Henke and J. W. M. DuMond, Submicroscopic Structure Determination by Long Wavelength X-Ray Diffraction, J. Appl. Phys. **26**, 903 (1955).

[7] J. Kirz, C. Jacobsen, and M. Howells, Soft X-ray microscopes and their biological applications, Q. Rev. Biophys. **28**, 33–130 (1995).

[8] A. Guinier, G. Fournet, C. B. Walker, and K. L. Yudowitch, *Small-angle Scattering of X-rays*, Structure of matter series (Wiley, New York, London, 1955).

[9] M. L. Klein and J. A. Venables, Rare gas solids Vol. II, Academic Press London (1977).

[10] P. Walter, T. Osipov, M.-F. Lin, *et al.*, The time-resolved atomic, molecular and optical science instrument at the Linac Coherent Light Source, J. Synchrotron Radiat. **29**, 957 (2022).

[11] K. R. Ferguson, *Crystal structure determinations of xenon nanoparticles and X-ray induced transient lattice contraction in the solid-to-plasma transition*, Ph.D. thesis, Stanford University (2016).

[12] T. M. Inc., MATLAB version: 23.2.0 (R2023b) (2023).

[13] L. Young, E. P. Kanter, B. Kräsignssig, *et al.*, Femtosecond electronic response of atoms to ultra-intense X-rays, Nature **466**, 56 (2010).

[14] T. Gorkhover, M. Adolph, D. Rupp, *et al.*, Nanoplasma dynamics of single large xenon clusters irradiated with superintense X-ray pulses from the linac coherent light source free-electron laser, Phys. Rev. Lett. **108**, 1 (2012).

[15] T. Gorkhover, S. Schorb, R. Coffee, *et al.*, Femtosecond and nanometre visualization of Struct.

Dyn. in superheated nanoparticles, Nat. Phot. **10**, 93 (2016).

[16] D. Rupp, L. Flückiger, M. Adolph, *et al.*, Imaging plasma formation in isolated nanoparticles with ultrafast resonant scattering, Struct. Dyn. **7**, 034303 (2020).

[17] J. Duris, S. Li, T. Driver, *et al.*, Tunable isolated attosecond X-ray pulses with gigawatt peak power from a free-electron laser, Nat. Phot. **14**, 30 (2020).
