# Supplementary material for: Nonlinear reversal of photoexcitation on the attosecond time scale improves ultrafast X-ray diffraction images
Source: Nat Commun. 2026 Jul 28;17:7556. doi: 10.1038/s41467-026-75969-8 (PMC13415538; doi:10.1038/s41467-026-75969-8)
Supplement: Supplementary file 2 — Transparent Peer Review file [file 41467_2026_75969_MOESM2_ESM.pdf]

nature portfolio

Peer Review File

Nonlinear reversal of photo-excitation on the attosecond time scale improves ultrafast x-ray diffraction images

Corresponding Author: Professor Tais Gorkhover

This file contains all reviewer reports in order by version, followed by all author rebuttals in order by version.

Version 0:

Reviewer comments:

Reviewer #1

(Remarks to the Author)
Thank you for the opportunity to review this manuscript “Nonlinear reversal of photo-excitation on the attosecond time scale improves ultrafast x-ray diffraction images” by Ulmer et al. The manuscript presents an interesting and valid experiment measuring the time dependent absorption cross section and x-ray scattering efficiency of neon nanoclusters at an XFEL. This work represents a continuation in a series of similar experiments from the group and collaborators from the past decade (see references [12], [15], [17-19], [23-24], [46], among others). This work, while exciting and valid has many similarities to reference [46] and thus the novelty of the current work is in question in my mind. Because of this and the other concerns at this time, I do not recommend it be published in Nature Communications. However, if the authors can appropriately address the concerns I would be happy to review the article again.

Major Concerns/Questions:
1) The current work and reference [46] are very similar experiments comparing the scattering efficiencies and cross sections of noble atom cryogenic clusters. Reference [46] studied Xe clusters and the current work Ne clusters but both discuss through experiment and simulation the effects of transient references. While this does not necessarily remove the novelty, it does seem lessen it and the impact should be described more in the introduction for the reader for a broad audience and high impact journal like Nature Communications.
2) The exact fluence used for the sub-fs pulses and the 15 fs pulses is not clear in the text as it says on page 4 ‘The x-ray fluence was similar for both pulse durations.’ Can you please clarify this and if possible describe how similar the two different pulses were in energy (give values or ranges in the main body) as well as details about average fluence and range of fluences for each pulse length? Even after reading the supplement and looking at Table I, it is not clear which of the two SASE situations were used in the 15 fs studies in the main body. Please clarify and seek for consistency in description of these important parameters, especially if the 15 fs pulses had 10 times the energy (62 micro-J compared to 600 micro-J)!
3) The evidence of stimulated emission is very exciting, would this be stimulated emission of the scattered photons or the unscattered photons (direct beam)? Is there a way to see this evidence of stimulated emission in the x-ray signal directly? This should be discussed in more detail.

Minor concerns/suggestions:
1) On page 3 at the top, you state electronic bleaching. Could you please provide a brief definition for the broader audience of Nature Communications?
2) On page 3 near the bottom, you state “We also find that partial x-ray excitation reversal through stimulated emission significantly increases the brightness of the images.” Can you please provide references or additional description about this claim?
3) Can you please provide an estimate of the number of Ne atoms in an average cluster, say of 80 nm diameter? This will help the reader in understanding the Q average ionization fraction.
4) On page 5 and Fig. 2 caption, it is mentioned that ‘eight’ of the brightest diffraction patterns are shown, however I only count 4 (2 in panel ‘b’ and 2 in panel ‘c’). Am I mistaken? If so, this needs to be clarified in the text and the caption.
5) Also in Fig. 2, is it possible to label a few of the vertical lines in the time of flight figures to show the
6) In Fig. 3 please label the y axis as “Q (average charge state per atom)” and also the 3.5 is missing off the E_res y axis, please add.
7) In Fig. 3 caption, there is a typo ‘the reschosonance’
8) On page 25 at the bottom, you say “the neutral case is the black curve” in Fig. 5, but it appears the line is actually purple,

is that just a display issue?
9) In the supplement on page 26, you reference “In Figure 5, we plot the results of a thought experiment, where we increase the FEL pulse energy ….” I believe you mean Fig. 6, not Fig. 5.

Reviewer #2

(Remarks to the Author)
The manuscript by A. Ulmer and colleagues reports the systematic investigation of scattering signal enhancement when using attoseconds X-ray pulses on core-level binding energy of the Ne atom. The signal enhancement was attributed to the stimulated emission effect with the short X-ray pulses outpacing the Auger decay time, and virtually launching repeating Rabi-oscillation type transition consecutively. The speculation was supported by performing experiments using slight longer pulse X-ray (15 fs), and on and off resonance experiments. Further MC simulation was performed to understand highly excited sstates induced by the intense, short pulse X-rays to convince the charge state and induced stimulated emission process. Overall, the work worths publication in the target journals with the following comments to be implemented properly.

1. Most importantly, but without any description in the manuscript, the spectral broadening (or poorly defined temporal coherence) is unavoidable in the short pulse radiation. Several to a few tens eV spectral broadening is expected in the present case for nominal ~ 870 eV resonance experiment. Knowing that this spectral broadening is not negligible, proper description/interpretation on how much this board banded incoming X-rays will give rise to an adversing effect on the stimulated resonance is essential. Large incoherent process is expected to induced with the sharp resonance transition buried within broad off-resonance ones.

2. The transient resonance, as a key concept of the current work also, was nicely introduced through the demonstration using Xe atoms before by the same group members. A progress is noted surely, but it is also essential to clearly state what is the new progress from the present work compare to the published work before (REF46). Certainly increased radial wave function overlap with the short pulse is definitely expected from the current work as well (as reported before in ref 46), but the authors attributes enhanced scattering cross section to the stimulated emission more in the present case. I think both effects (along with possible others) can be involved. In any case, clear comparison analysis would be essential to highlight the novelty of the present discovery.

3. Descriptions on how the scattering cross section was estimated from the diffraction signal; how did the same size effect is called/considered properly, how is the incident photon flux is calibrated, given the small sample size the exact hit position of the sample with respect to the indecent X-ray beam (dominantly along the transverse direction) is properly considered before quantifying the scattering cross section, etc. are necessary on the main manuscript. Good details are given only in the supplementary information. I think some part can be moved to the main text to make the manuscript more readable by itself. Also, some information on the technical issues of above mentioned needs to be described.

4. Some casual expressions are noted when describing key features to confuse the readers to understand important messages. They include for instance 'excitation/absorption inside the NP is reversed' --> r'eversed' intend to say what? ; 'remain longer resonant' --> 'longer resonant' means what? on page 7. There are several more in the manuscript and those are better be described more explicitly and scientifically. Also many typing errors are noted.

5. The authors always seemed to have witnessed 0.6 charge state difference between sub-fs and 15 fs illumination (Fig. 2). It appears fairly unnatural/arbitrary but no description on it was made.

6. (Results on Fig. 2) The scattering cross section on ground state Ne displays a resonance several eV before the main deep from the K-edge. Where does this feature come from? Also on (c), the scattering intensity from 15fs seemed to be larger than sub-fs one.

7. (Fig. 3 related) The number of scattered photons seemed to show saturated behavior (dominantly above the edge experiment) irrespective of the charge states. Physically it sounds odds, and it would be good to see some description on it in the manuscript.

Reviewer #3

(Remarks to the Author)
Dear Editor,
The authors discuss how sub-fs X-ray pulses enhance scattering cross sections and diffraction images while at the same time resulting in a lower charge state for the case of Neon nanoparticles. They show through simulations that this is due to stimulated emission.

Overall, I find the paper very interesting. However, I believe that in the current version certain things in the manuscript are not clear and need to be explicitly addressed by the authors.
1) I believe that, already in the introduction, the authors should make absolutely clear what they have found that is new and has not been observed before.
They make some comparisons with past literature in the rest of the manuscript.
However, in my opinion this is not sufficient. Clear comparisons should be made already in the introduction and should be clearly stated how their work differs compared to previous work. For instance, in page 5, end of first paragraph, they mention

previous studies on Xe nanoparticles. A clear comparison should be made already in the introduction, i.e. what were the findings for the Xe nanoprtciles and how they differ with the current ones for Ne. This becomes clear only later after reading the whole paper. Also, in page 6, just before the Modeling section, the authors mention that “The Xe nanoparticle study was recorded with significantly lower FEL fluences in sub-fs FEL pulses, thus the influence of nonlinear effects was overall less prominent”. Which nonlinear effects do they refer to? Are these non-linear effects present in their current study?
In addition, in the outlook section, the authors state that “Rabi oscillations in Ne+ in the soft X-ray regime have been predicted in the past …”. So what is the difference from their study? Just that they employ hard X-rays in the current study? All these should be clarified and mentioned in the introduction, so that it becomes very clear what is new in this study.
2) The authors state that “our simulation explains this behavior through two effects. First, sub-fs pulses outrun the Auger decay processes and most nanoplasma formation effects”, while the second effect is stimulated emission. I have no issue with the stimulated emission and indeed the authors have shown with Fig.4 how stimulated emission enhances scattering cross sections near the resonance. However, my objection is the first effect. Besides, the obvious fact that sub-fs X-rays are shorter than the femtosecond scale of Auger rates, I do not see what the authors have done in their simulations that actually demonstrate this. If I am missing something, I would appreciate it if the authors clarify and specifically explain what part of their simulation demonstrates the first effect.

Second, I find that in places in the manuscript it is not clear what the authors
mean.
• Page 2, first paragraph, when the authors write “dose” do they refer to intensity? If not, I think the authors should specify exactly what they mean.
• Same paragraph, when the authors write “is a hard limit to the signal-to-noise ratio of the image” it is not clear to me to what does the word “noise” refer to. From what the authors write above, I understand that the authors discuss that the main issue with high intensity X-ray pulses is that they can cause complete photo-ionization, with no electrons remaining. If this is correct, I do not see how the word “noise” fits in.
• Page 3, 2nd paragraph, the authors write that “transient resonances can increase the scattering cross sections beyond literature values”. These values refer to absorption by FEL pulses? By single photon absorption? The authors should specify and cite the relevant papers.
• Same paragraph, the authors also write “partial x-ray excitation reversal through stimulated emission significantly increases the brightness of the images”. My understanding from reading the rest of the paper is that this is the case for the sub-fs pulse. If this is the case then this should be specifically mentioned.
• Page 4, second paragraph, by “Single-particle” do the authors mean “Single-nanoparticle”? If so, they have to write so to be consistent with the rest of the manuscript.
• Page 4, fourth paragraph, I do not understand the “Each dot, corresponds to a single image”, for Figure 2. It is a single image following from diffraction from all nanoparticles in contrast to Fig. 3 where each dot follows from diffraction from a single nanoparticle?
• Same paragraph, the authors should reference the “literature values for Ne atoms” concerning cross sections.
• Page 5, 2nd paragraph, it is not clear what the authors mean when they write “Q does not reflect the exact average charge during the exposure but is rather a relative measure of the total absorbed energy per atom”. Do they refer to the fact that Q varies over exposure since it can be higher and then lower due to stimulated emission ? If so, then maybe this sentence should come later in the manuscript or they should clearly relate it to stimulated emission that is discussed later in the paper.
• There is a typo for “resonances” in the caption of Fig.3.

Next, I found several issues with the Supplementary section.

1) The main issue is that currently the Theory and Experimental Details sections seem to be written in a non consistent way. To be clear, I mean that quantities that appear first in the Theory section are never defined in this section but then are defined later in the Experimental Details. The authors need to write consistently these sections so that quantities are defined already in the Theory section which is what one reads first. For instance, n_{a} is not defined in Theory but is defined in the Experiment part, f^{0}(\omega) is not discussed at all in Theory but shows up in Eq. 19 of the Experiment section.
2) Page 23, the authors mention after Eq. 1 a molecular dynamics algorithm which is used. They should cite this algorithm.
3) It is not clear to me what is the difference between the “pulse fluence j_{x} and the fluence of the XFEL pulse in page 23. Can the authors explain?
4) Page 24, in Eq. 4 how do the authors find the form factors? Is there a reference?
5) Page 25, 2nd paragraph, the authors write, “the amplification of diffraction can go up to 6 orders of magnitude” when discussing Fig. 5. The authors should explain clearly in what part of the figure we can find these change of 6 orders of magnitude.
6) The authors refer to a phase shift between quasi-free and bound electrons that can possibly explain differences between theory and experiment. Can the authors explain how this shift enters (if it does) the Eqs the authors list in the Theory section? In my opinion, as it currently stands it is not clear what they mean.
7) The authors should correct the colors in Fig. 5 and 6. In page 25, they say that “The neutral case is the black curve” but in Figure 5 it seems to be the purple curve. In Fig. 6 caption, again for neutral Ne they write “blue solid line” but it is actually the purple line.
8) Page 26, they write “In Fig. 5 we plot….from 0.05mJ to 0.6 mj”. I believe it is Fig.6 and it is from 0.06 mJ.

Concluding, I find this work to be very interesting. However, it needs to address the issues I mention above, i.e. consistent and careful writing of the supplemental material and mostly clearly stating in the introduction what is new compared to previous studies, so the reader can clearly assess the novelty of the work.

Sincerely, Agapi Emmanouilidou.

Version 1:

Reviewer comments:

Reviewer #1

(Remarks to the Author)
After reviewing the revised article 'Nonlinear reversal of photo-excitation on the attosecond time scale improves ultrafast x-ray diffraction images' I see that all of my concerns have been addressed. The majorly revised introduction and discussion/conclusion sections greatly increase the applicability and impact of the current work. The authors have put the current work in light with the important past work by this group and others. They have also described clearly how this is the first demonstration for them of x-ray sub-fs experiments instead of previous XUV or soft x-ray. The demonstration of simulated emission and controlled damage opens a very interesting and powerful method. I believe the article should now be published in Nature Communications.

Reviewer #2

(Remarks to the Author)
The authors have properly revised the manuscript by incorporating the points raised in the original version. Important messages, some descriptions are left to the SI, should still be delivered in the mani text rather than SI, as a minor comment, which is optional.
I think the manuscript is ready for the publication.

Reviewer #3

(Remarks to the Author)
Dear Editor,
I believe that the authors have answered and addressed all the reviewers' comments.
Therefore, I suggest publication of this work to Nature Commun.
Best Regards, Agapi Emmanouilidou.

Open Access This Peer Review File is licensed under a Creative Commons Attribution 4.0 International License, which permits use, sharing, adaptation, distribution and reproduction in any medium or format, as long as you give appropriate credit to the original author(s) and the source, provide a link to the Creative Commons license, and indicate if changes were made.
In cases where reviewers are anonymous, credit should be given to 'Anonymous Referee' and the source.
The images or other third party material in this Peer Review File are included in the article's Creative Commons license, unless indicated otherwise in a credit line to the material. If material is not included in the article's Creative Commons license and your intended use is not permitted by statutory regulation or exceeds the permitted use, you will need to obtain permission directly from the copyright holder.
To view a copy of this license, visit https://creativecommons.org/licenses/by/4.0/

REVIEWER COMMENTS

First of all, we would like to thank the reviewers for their thorough reviews, excellent comments and great feedback. Reading the reviews we realized that some important details in the paper were presented not clear enough. More importantly, we recognized that we need to emphasize and explain more our main and surprising finding, that stimulated emission can partially reverse X-ray damage and increase the diffraction brightness in parallel. Based on the comments from all referees we have substantially changed the introduction and discussion and extended the supplemental material accordingly.

We are very encouraged that all referees stress the relevance of the presented work, including "… the evidence of stimulated emission is very exciting…", "… the work is worths publication in the target journal…", to "… I find the paper very interesting…".

We have followed the comments from the referees as detailed below. We believe that the many and very good comments have greatly helped us improving the manuscript. We firmly believe the new manuscript shows both, the power and potential of intense sub-fs X-ray pulses for imaging and non-linear spectroscopy of complex systems.

Reviewer #1 (Remarks to the Author):

Thank you for the opportunity to review this manuscript "Nonlinear reversal of photo-excitation on the attosecond time scale improves ultrafast x-ray diffraction images" by Ulmer et al. The manuscript presents an interesting and valid experiment measuring the time dependent absorption cross section and x-ray scattering efficiency of neon nanoclusters at an XFEL. This work represents a continuation in a series of similar experiments from the group and collaborators from the past decade (see references [12], [15], [17-19], [23-24], [46], among others). This work, while exciting and valid has many similarities to reference [46] and thus the novelty of the current work is in question in my mind. Because of this and the other concerns at this time, I do not recommend it be published in Nature Communications. However, if the authors can appropriately address the concerns I would be happy to review the article again.

We would like to thank the reviewer for a thorough and constructive review.

Major Concerns/Questions:
1) The current work and reference [46] are very similar experiments comparing the scattering efficiencies and cross sections of noble atom cryogenic clusters. Reference [46] studied Xe clusters and the current work Ne clusters but both discuss through experiment and simulation the effects of transient references. While this does not necessarily remove the novelty, it does seem lessen it and the impact should be described more in the introduction for the reader for a broad audience and high impact journal like Nature Communications.

Thank you for highlighting the need to better position our results within the existing literature. The central novelty of this work is the identification of stimulated emission as a powerful control parameter for tailoring the refractive index and, remarkably, for reversing X-ray–induced damage. We have changed a significant part of the introduction to:

"… Over the past decade, multiple studies spanning the XUV to the hard X-ray regime have shown that nonlinear effects, such as transient resonances, can substantially accelerate photoionization and, in consequence, electronic structure degradation~\cite{Bostedt2012,Rupp2020,Kanter2011,Rudek2012,Rudek2018relativistic}. When these resonances occur ions formed by X-rays can absorb much more strongly than neutral atoms. In a previous study on Xe nanoparticles, we have demonstrated that transient resonances can amplify soft X-ray small angle diffraction obtained with 10 fs short pulses beyond reported literature values~\cite{NIST} before the onset of atomic structural disintegration ~\cite{Kuschel2025}. However, we have also observed that transient resonances significantly accelerate electronic damage and the sample destruction due to increased absorption cross-sections. Thus, longer exposures (>100 fs) resulted in a significant reduction of the scattering cross-section. This observation is consistent with earlier work on lighter-element nanoparticles (e.g., sucrose nanospheres), where 150 fs pulses led to reduced diffraction yield and accelerated structural degradation, both linked to transient resonances in oxygen ions~\cite{Ho2020}. Collectively, these findings established the prevailing view that the principal advantage of shorter pulses simply lies in their ability to outrun atomic motion. While transient resonances may increase scattering cross-sections in short-lived electronic states, these resonances accelerate electronic bleaching, which remains largely irreversible.

Here, we demonstrate that newly available intense sub-femtosecond FEL pulses fundamentally challenge this picture. They provide access to a transiently resonant regime in which stimulated emission can be exploited, enabling reversal of photoabsorption while preserving enhanced scattering cross-sections at extreme X-ray power densities approaching 10^{20} W/cm^2. Rather than merely outrunning damage, our results point to active control of the refractive index and damage in dense and highly excited nanosystems. …"

2) The exact fluence used for the sub-fs pulses and the 15 fs pulses is not clear in the text as it says on page 4 'The x-ray fluence was similar for both pulse durations.' Can you please clarify this and if possible describe how similar the two different pulses were in energy (give values or ranges in the main body) as well as details about average fluence and range of fluences for each pulse length? Even after reading the supplement and looking at Table I, it is not clear which of the two SASE situations were used in the 15 fs studies in the main body. Please clarify and seek for consistency in description of these important parameters, especially if the 15 fs pulses had 10 times the energy (62 micro-J compared to 600 micro-J)!

Based on this comment, we have fully removed the mentioning of the unattenuated 15 fs pulses as they are not relevant for the observations. Also, we have added to the main text:

"…The average pulse energy for 15 fs pulses was 110 µJ and for sub-fs pulses 60 µJ. The pulse energy distribution for sub-fs pulses was skewed towards higher pulse energies. In order to match those intense pulses, we chose to slightly increase the average pulses energy of the 15 fs pulses (see Supplementary Table 1 and Supplementary Fig. 4 for details)..."

3) The evidence of stimulated emission is very exciting, would this be stimulated emission of the scattered photons or the unscattered photons (direct beam)? Is there a way to see this evidence of stimulated emission in the x-ray signal directly? This should be discussed in more detail.

Thank you for sharing our excitement about the observed effects from the stimulated emission! These are important questions. A rigorous quantum electrodynamics (QED) treatment of stimulated emission, resonant fluorescence, and Rabi oscillations in a two-atom system has recently been developed by our co-author Phay Ho (https://oviv.org/pdf/2506.06585).

Our data indicates that the complex refractive index can be significantly influenced by stimulated emission. In a macroscopic description based on Maxwell's equations, stimulated emission can be treated as negative absorption, corresponding to a negative imaginary part of the refractive index. In this framework, stimulated emission enhances the coherent polarization and contributes to the scattered field at all angles, not only to the forward (unscattered) beam. In our experiment, this affects mostly the brightness of the image. For higher rates of stimulated emission, one could expect that the diffraction pattern shape could change. We have added to the "Discussion" part of the main text:

"…In a macroscopic description based on Maxwell's equations, stimulated emission can be regarded akin to negative absorption, which is represented by the imaginary part of the refractive index. Thus, one can assume that stimulated emission will modify the scattered field beyond the forward direction. In the present experiment, this contribution mostly affects the brightness of the image. However, when stimulated emission occurs at higher rates, the angular distribution of scattered intensity might change, analogous to the role of absorption and dispersion in Mie scattering. Such effects would offer new opportunities for enhancing material contrast…"

Minor concerns/suggestions:
1) On page 3 at the top, you state electronic bleaching. Could you please provide a brief definition for the broader audience of Nature Communications?

We have changed the text in the manuscript´s Introduction to:

"…A key limitation of this approach remains the electronic bleaching of the sample [4, 10, 25–27]. Intense X-ray pulses rapidly deplete electronic ground states within femtoseconds through excitation, photoionization, and subsequent relaxation cascades. In extreme cases, many electrons are removed from the parent ions and atoms become effectively transparent to the probe before significant atomic motion occurs. Such electronic bleaching or damage is regarded as the ultimate limit for the diffraction signal in high scattering angles and thus, to the spatial resolution in X-ray diffraction imaging. Electron bleaching also affects the sensitivity and accuracy in spectroscopic studies, especially for site-specific nonlinear schemes in complex specimen [12, 23, 25, 27–34].

2) On page 3 near the bottom, you state "We also find that partial x-ray excitation reversal through stimulated emission significantly increases the brightness of the images." Can you please provide references or additional description about this claim?

We revised the wording of the claim that is based upon results presented in this paper "…"Stimulated emission drives excited electrons back into a transient resonant state associated with an enhanced scattering cross section."…"

3) Can you please provide an estimate of the number of Ne atoms in an average cluster, say of 80 nm diameter? This will help the reader in understanding the Q average ionization fraction.

At the focus center, every atom is ionized and Q is defined as the average charge state per atom. Thus, Q does not depend on the size of the nanoparticle in our experiment. However, the atom number gives a good estimate for the complexity of the sample, we have added to page 5: "…with diameters of 60 nm to 100 nm (0.5 × 10^7 to 2.2 × 10^7 atoms)…"

4) On page 5 and Fig. 2 caption, it is mentioned that 'eight' of the brightest diffraction patterns are shown, however I only count 4 (2 in panel 'b' and 2 in panel 'c'). Am I mistaken? If so, this needs to be clarified in the text and the caption.

Thank you for pointing out the unclear explanation. Fig 2 was described in the main text and the shots are highlighted in the Figure 3, but the averaging was not clearly described in the caption to Figure 2. The diffraction pattern displayed are averages of the 8 brightest shots, which are marked as bold dots in Figure 3. We have added to the Fig. 2 caption:

"… In panel (b) and (c), the average of the eight brightest diffraction patterns is displayed on the left side for photon energies 866 eV and 956 eV. On the right side, the corresponding simultaneously recorded ion time-of-flight spectra are shown…"

5) Also in Fig. 2, is it possible to label a few of the vertical lines in the time of flight figures to show the *Done*

6) In Fig. 3 please label the y axis as “Q (average charge state per atom)” and also the 3.5 is missing off the E_res y axis, please add. *Done*

7) In Fig. 3 caption, there is a typo ‘the reschosonance’ *Done*

8) On page 25 at the bottom, you say “the neutral case is the black curve” in Fig. 5, but it appears the line is actually purple, is that just a display issue? *Purple is correct, thanks! Done*

9) In the supplement on page 26, you reference “In Figure 5, we plot the results of a thought experiment, where we increase the FEL pulse energy ….” I believe you mean Fig. 6, not Fig. 5. *Yes, done.*

Reviewer #2 (Remarks to the Author):

The manuscript by A. Ulmer and colleagues reports the systematic investigation of scattering signal enhancement when using attoseconds X-ray pulses on core-level binding energy of the Ne atom. The signal enhancement was attributed to the stimulated emission effect with the short X-ray pulses outpacing the Auger decay time, and virtually launching repeating Rabi-oscillation type transition consecutively. The speculation was supported by performing experiments using slight longer pulse X-ray (15 fs), and on and off resonance experiments. Further MC simulation was performed to understand highly excited sstates induced by the intense, short pulse X-rays to convince the charge state and induced stimulated emission process. Overall, the work worths publication in the target journals with the following comments to be implemented properly.

Thank you for this positive perspective on our work and your thoughtful comments.

1. Most importantly, but without any description in the manuscript, the spectral broadening (or poorly defined temporal coherence) is unavoidable in the short pulse radiation. Several to a few tens eV spectral broadening is expected in the present case for nominal ~ 870 eV resonance experiment. Knowing that this spectral broadening is not negligible, proper description/interpretation on how much this board banded incoming X-rays will give rise to an adversing effect on the stimulated resonance is essential. Large incoherent process is expected to induced with the sharp resonance transition buried within broad off-resonance ones.

This is an important point, and we agree that the finite spectral bandwidth of sub-fs FEL pulses must be carefully considered when interpreting stimulated emission. We added a new section to the Supplements with the following discussion of the bandwidth:

"…Supplementary Note 5.c: Influence of sub-fs FEL pulse bandwidth on stimulated emission

In our experiment, the measured FEL bandwidth is approximately 6 eV (see Supplementary Table 1), which is consistent with previous measurements of sub-fs FEL pulses reported by Duris et al. [40]. While this bandwidth is not negligible, its impact on stimulated emission depends critically on the structure and separation of the relevant resonances. There are two key aspects to consider.

First, despite the finite bandwidth, the ionic resonances remain well resolved and pronounced. This is demonstrated in Supplementary Fig. 1(b), where the calculated response including the measured FEL bandwidth still shows clear resonant features. This occurs because the relevant core–excited ionic transitions are separated in energy by amounts mostly larger than the FEL bandwidth.

Second, although broadband excitation can in principle enhance incoherent processes, particularly for neutral Ne where the 1s–3p transition lies close to the continuum, our simulations show that stimulated emission from neutral Ne is negligible under our experimental conditions. This result is physically expected because, at the highest FEL intensities used here, each Ne atom absorbs on average more than 2–5 photons per pulse, leading to rapid ionization and depletion of the neutral population. The dominant contribution to stimulated emission in our experiment instead arises from ionic species, primarily Ne2+, Ne3+,and Ne4+, involving 1s–2p and 1s–3p transitions. In these ions, the excited states are well separated from the continuum and from neighboring excited levels. For example, in Ne2+ the 3p level lies approximately 25 eV below the continuum, while the next higher 4p level is separated by about 10 eV. Both energy separations exceed the FEL bandwidth. As a result, these transitions can be treated to a good approximation as isolated two-level systems, for which stimulated emission remains efficient even in the presence of finite spectral bandwidth.

We note that nanoplasma effects, such as ionization potential depression, can shift resonance energies and modify transition strengths. However, these shifts are expected to remain comparable to or smaller than the level spacings discussed above and therefore do not qualitatively change our conclusion that the relevant ionic resonances remain spectrally distinguishable within the FEL bandwidth. Furthermore, the ultrashort sub-fs pulse duration ensures that stimulated emission occurs on a timescale comparable to or faster than competing incoherent processes such as impact ionization and Auger decay, which become more significant only for longer (few-fs) pulses. In summary, although the FEL bandwidth is finite, it remains sufficiently narrow compared to the relevant ionic level separations to preserve the resonant character of the transitions responsible for stimulated emission…"

2. The transient resonance, as a key concept of the current work also, was nicely introduced through the demonstration using Xe atoms before by the same group members. A progress is noted surely, but it is also essential to clearly state what is the new progress from the present work compare to the published work before (REF46). Certainly increased radial wave function overlap with the short pulse is definitely expected from the current work as well (as reported before in ref 46), but the authors attributes enhanced scattering cross section to the stimulated emission more in the present case. I think both effects (along with possible others) can be involved. In any case, clear comparison analysis would be essential to highlight the novelty of the present discovery.

Thank for pointing out the necessity to clarify the role of stimulated emission compared to the previous studies. This point has been brought up by all three referees and we have changed the introduction accordingly (please see the entire page 3 and the first paragraphs on page 4). Moreover, we have added to the description of our results on page 7:

"…Near the Ne K-edge, we observe in Fig. 2 and Fig. 3 that sub-fs FEL pulses are absorbed less efficiently but simultaneously produce stronger diffraction than few-femtosecond FEL pulses with a comparable fluence. This diverging behavior is unexpected in light of previous experimental studies. Earlier work in the XUV and soft X-ray regimes demonstrated enhanced absorption due to transient resonances [17, 19, 32, 35, 36, 39, 52]. These experiments supported by simulations, showed that transient resonances increase absorption and thereby accelerate electronic excitation and ionic damage in the sample for all pulse durations. In contrast, the present results indicate a qualitatively different regime. Compared to the first reported sub-fs soft X-ray diffraction experiments (Kuschel et al.), the sub-fs FEL pulses used here deliver up to an order of magnitude higher pulse energy at the sample. This substantial increase in fluence is critical for accessing nonlinear light–matter interaction regimes, where processes such as stimulated emission can modify the net absorption throughout the pulse.

3. Descriptions on how the scattering cross section was estimated from the diffraction signal; how did the same size effect is called/considered properly, how is the incident photon flux is calibrated, given the small sample size the exact hit position of the sample with respect to the indecent X-ray beam (dominantly along the transverse direction) is properly considered before quantifying the scattering cross section, etc. are necessary on the main manuscript. Good details are given only in the supplementary information. I think some part can be moved to the main text to make the manuscript more readable by itself. Also, some information on the technical issues of above mentioned needs to be described.

To address these concerns, we have added to the main text more details concerning the fluence (see page 5 and 6):

"… The average pulse energy for 15 fs pulses was 110 µJ and for sub-fs 60 µJ. The pulse energy distribution for sub-fs pulses was skewed towards higher pulse energies. In order to match those intense pulses, we chose to slightly increase the average pulses energy of the 15 fs pulses (see Supplementary Table 1 and Supplementary Fig. 4 for details) …"

"…Single-exposure X-ray diffraction snapshots of nanoparticles are recorded at 60 Hz using the ePix detector [49], which is located 0.395 m downstream from the interaction region. The particle size is encoded directly into Airy pattern-like diffraction patterns and can be recovered with 0.3 nm accuracy [21, 50]…"

"…The measured variation of the absorbed energy and the diffraction cross-sections across the photon energy scan is exhibited in Fig. 2. From each individual diffraction pattern, we extracted the forward scattering intensity per Ne atom inside the nanoparticle. The average atomic scattering cross-section can then be extracted by normalizing the scattering intensity to the incident FEL pulse fluence and, similar to previous studies, by assuming that the brightest of the normalized scattering intensities were produced near the FEL focus center [18, 32, 39]. The FEL pulse fluence is deduced from the pulse energy recorded shot-to-shot by an inline gas monitor. For details on the calculation see Supplementary Notes 3 and 4. The fitting routine for the nanoparticle size and shape is described in Supplementary Note 5.a. In Fig. 2 (a), the scattering cross-sections σ_{sca} extracted from the top 5% of the brightest normalized patterns are plotted against the incoming photon energy $\hbar\omega$. Each dot corresponds to a single image from an individual Ne nanoparticle…"

We have also structured the references to the Supplements in a more systematic fashion.

4. Some casual expressions are noted when describing key features to confuse the readers to understand important messages. They include for instance 'excitation/absorption inside the NP is reversed' --> r'eversed' intend to say what? ; 'remain longer resonant' --> 'longer resonant' means what? on page 7. There are several more in the manuscript and those are better be described more explicitly and scientifically. Also many typing errors are noted.

We have changed all these descriptions to more detailed explanations throughout the manuscript mainly in the aforementioned Introduction and Discussion.

5. The authors always seemed to have witnessed 0.6 charge state difference between sub-fs and 15 fs illumination (Fig. 2). It appears fairly unnatural/arbitrary but no description on it was made.

This is an interesting observation, which we did not notice previously. Our simulation displayed in Fig 5., right side indicates that for photon energies 866 eV and 956 eV indeed the average charge state difference is very similar at the end of the exposure (ΔQ =1.3 @ 866 eV compared to ΔQ =1.25 eV 956 eV difference). It is reasonable to

assume that even after significant recombination and cooling of the plasma, the difference in average charge state will remain similar. Thus, this experimental finding further strengthens our modeling. We have added a comment to Figure 4 caption:

"… Similar to our experimental observation, the average charge state difference between both pulse durations at 866 eV and 956 eV are similar (around two) …"

6. (Results on Fig. 2) The scattering cross section on ground state Ne displays a resonance several eV before the main deep from the K-edge. Where does this feature come from? Also on (c), the scattering intensity from 15fs seemed to be larger than sub-fs one.

(a) This is the 1s-3p pre-edge resonance, the same feature can be found in NEXAFS experimental data and in reference Rohringer2008resonant. (c) Yes, that is correct. Here, due to the Auger decay and impact ionization, the average state is greater during the pulse. Higher charge states have resonances at higher photon energies, therefore there are transient resonances which the longer pulses can access but the sub-fs pulses cannot. We have added to the Fig.2 caption:

(a)"…The pre-edge transitions lead to a small maximum before the edge, which is broadened due to a convolution with a 6 eV Gaussian profile (FWHM) to account for the bandwidth of the FEL…"

(b)"… The 15 fs pulses appear to generate brighter images at 956 eV. This hits to higher charge state generation, which has binding energies tens of eVs above the edge…"

7. (Fig. 3 related) The number of scattered photons seemed to show saturated behavior (dominantly above the edge experiment) irrespective of the charge states. Physically it sounds odds, and it would be good to see some description on it in the manuscript.

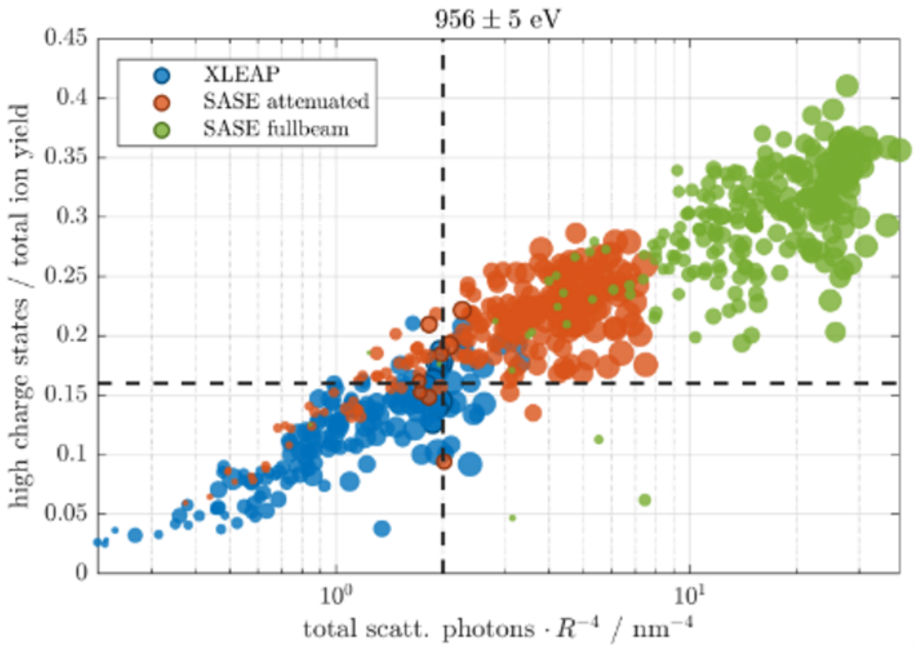

Maybe the referee is referring to the bold dots, which represent the top 5% of images used in Figure 2. They might create the illusion of a saturation. On the left side is the same data and in addition are green dots from the 15 fs pulses without attenuation (roughly 4-5 more photons per pulse than the attenuated red dots).

There is always some spread on the measured average charge states, but the general trend (higher scattering cross section increases with the average charge state) seems to continue even for significantly more intense FEL pulses.

Based on other comments, we have decided to remove the mentioning of the unattenuated beam 15 fs pulses from the paper in order to avoid confusion about the fluences used for the comparison with sub-fs pulses. The unattenuated beam data does not affect any messages of the paper. For clarity, we would prefer to leave it out of this paper. However, if the referee thinks otherwise, we can add this graph to the Supplements.

Reviewer #3 (Remarks to the Author):

The authors discuss how sub-fs X-ray pulses enhance scattering cross sections and diffraction images while at the same time resulting in a lower charge state for the case of Neon nanoparticles. They show through simulations that this is due to stimulated emission.

Overall, I find the paper very interesting. However, I believe that in the current version certain things in the manuscript are not clear and need to be explicitly addressed by the authors.

Thank you for your interest in our paper and carefully reviewing our paper.

1) I believe that, already in the introduction, the authors should make absolutely clear what they have found that is new and has not been observed before.
They make some comparisons with past literature in the rest of the manuscript. However, in my opinion this is not sufficient. Clear comparisons should be made already in the introduction and should be clearly stated how their work differs compared to previous work. For instance, in page 5, end of first paragraph, they mention previous studies on Xe nanoparticles. A clear comparison should be made already in the introduction, i.e. what were the findings for the Xe nanoprtciles and how they differ with the current ones for Ne. This becomes clear only later after reading the whole paper.
Thank you for pointing this out similar to other referees.

Thank you for pointing this out similar to other referees. Based on your comments, as well as similar suggestions from other reviewers, we have completely rewritten the introduction, which should now clarify the progress in the present study (see page 3 and 4).

Also, in page 6, just before the Modeling section, the authors mention that "The Xe nanoparticle study was recorded with significantly lower FEL fluences in sub-fs FEL pulses, thus the influence of nonlinear effects was overall less prominent". Which nonlinear effects do they refer to? Are these non-linear effects present in their current study?

To clarify these aspects, we have added to the main text on page 7: "…Near the Ne K-edge, we observe in Fig. 2 and Fig. 3 that sub-fs FEL pulses are absorbed less efficiently but simultaneously produce stronger diffraction than few-femtosecond FEL pulses with a comparable fluence. This diverging behavior is unexpected in light of previous experimental studies. Earlier work in the XUV and soft X-ray regimes demonstrated enhanced absorption due to transient resonances [17, 19, 32, 35, 36, 39, 52]. These experiments, supported by simulations, showed that transient resonances increase absorption and thereby accelerate electronic excitation and ionic damage in the sample for all pulse durations. In contrast, the present results indicate a qualitatively different regime. Compared to the first reported sub-fs soft X-ray diffraction experiments, the sub-fs FEL pulses used here deliver up to an order of magnitude higher pulse energy at the sample. This substantial increase in fluence is critical for accessing nonlinear light–matter interaction regimes, where processes such as stimulated emission can modify the net absorption throughout the pulse…"

In addition, in the outlook section, the authors state that "Rabi oscillations in Ne+ in the soft X-ray regime have been predicted in the past …". So what is the difference from their study? Just that they employ hard X-rays in the current study? All these should be clarified and mentioned in the introduction, so that it becomes very clear what is new in this study.

We are referring to a theoretical study for isolated, single atoms. We have modified the claim in the text on page 10 to:

"…In principle, the electronic state and X-ray induced damage can be coherently controlled via Rabi oscillations, enabled by the high temporal coherence of sub-fs FEL pulses. Rabi oscillations in isolated Ne+ ions have been suggested in the soft X-ray regime [35, 44] and observed experimentally in the XUV regime in He atoms [55, 56]. Extending this concept from dilute atomic gases to dense, highly excited nanoplasmas opens a pathway toward coherent control of the X-ray refractive index in structurally complex systems.…"

2) The authors state that "our simulation explains this behavior through two effects. First, sub-fs pulses outrun the Auger decay processes and most nanoplasma formation effects", while the second effect is stimulated emission. I have no issue with the stimulated emission and indeed the authors have shown with Fig.4 how stimulated emission enhances scattering cross sections near the resonance. However, my objection is the first effect. Besides, the obvious fact that sub-fs X-rays are shorter than the femtosecond scale of Auger rates, I do not see what the authors have done in their simulations that actually demonstrate this. If I am missing something, I would appreciate it if the authors clarify and specifically explain what part of their simulation demonstrates the first effect.

The main nanoplasma formation effect, which increases the charge state, is collisional ionization. We have added to the text on page 9:

"…In contrast, 15 fs pulses produce substantially higher charge states, typically between Ne6+ and Ne8+ (red dotted line), driven mainly by Auger relaxation cascades and nanoplasma formation. According to our simulation, collisional ionization produces more than 50% of the total charge state for longer pulses. The number of collisional ionization events per-atom is 4.4 for 815 eV and 4.9 at 877 eV for a 15-fs pulse…"

Second, I find that in places in the manuscript it is not clear what the authors mean.

• Page 2, first paragraph, when the authors write "dose" do they refer to intensity? If not, I think the authors should specify exactly what they mean.

Dose is now changed to photons per unit area.

• Same paragraph, when the authors write "is a hard limit to the signal-to-noise ratio of the image" it is not clear to me to what does the word "noise" refer to. From what the authors write above, I understand that the authors discuss that the main issue with high intensity X-ray pulses is that they can cause complete photo-ionization, with no electrons remaining. If this is correct, I do not see how the word "noise" fits in.

In diffraction imaging the signal will approach the shot noise in the higher scattering angles, thus limiting the resolution of higher spatial frequencies. While our expression is technically correct, we acknowledge that it might sound misleading without a ckear specification. We have modified the expression to:

"…Such electronic bleaching is regarded as the ultimate limit for the diffraction signal in high scattering angles and thus, to the spatial resolution in X-ray diffraction imaging. Electron bleaching also affects the sensitivity and accuracy in spectroscopic studies, especially for site-specific nonlinear schemes in complex specimen [12, 23, 25, 27–34] …"

• Page 3, 2nd paragraph, the authors write that "transient resonances can increase the scattering cross sections beyond literature values". These values refer to absorption by FEL pulses? By single photon absorption? The authors should specify and cite the relevant papers.

We refer to the NIST "X-ray Form Factor, Attenuation and Scattering Tables". This is now clarified in the paper.

• Same paragraph, the authors also write "partial x-ray excitation reversal through stimulated emission significantly increases the brightness of the images". My understanding from reading the rest of the paper is that this is the case for the sub-fs pulse. If this is the case then this should be specifically mentioned.

We have now specified that the sentence is about sub-fs exposures. We have added to the text in the Introduction:

"… Here, we demonstrate that newly available intense sub-femtosecond FEL pulses fundamentally challenge this picture. They provide access to a transiently resonant regime in which stimulated emission can be exploited, enabling reversal of photoabsorption while preserving enhanced scattering cross-sections at extreme X-ray power densities approaching 10^20W/cm2. Rather than merely outrunning damage, our results point to active control of the refractive index and damage in dense and highly excited nanosystems …"

• Page 4, second paragraph, by "Single-particle" do the authors mean "Single-nanoparticle"? If so, they have to write so to be consistent with the rest of the manuscript.

We have changed "Single-particle" to "Single-nanoparticle" or "individual nanoparticles" throughout the manuscript.

• Page 4, fourth paragraph, I do not understand the "Each dot, corresponds to a single

image", for Figure 2. It is a single image following from diffraction from all nanoparticles in contrast to Fig. 3 where each dot follows from diffraction from a single nanoparticle?

Each dot is always a single diffraction image from a single nanoparticle in Figure 2 (a) and Figure 3 (a)-(b). The diffraction images and the ion spectra demonstrated in Figure 2 on the left side, are averages of the 8 brightest diffraction patterns per condition. We have added to the main text:

"… Each dot corresponds to a single image from an individual Ne nanoparticle…"

We have added similar descriptions to the captions of Figure 2 and 3.

• Same paragraph, the authors should reference the "literature values for Ne atoms" concerning cross sections.

We refer to the NIST "X-ray Form Factor, Attenuation and Scattering Tables". This is now clarified in the paper.

• Page 5, 2nd paragraph, it is not clear what the authors mean when they write "Q does not reflect the exact average charge during the exposure but is rather a relative measure of the total absorbed energy per atom". Do they refer to the fact that Q varies over exposure since it can be higher and then lower due to stimulated emission ? If so, then maybe this sentence should come later in the manuscript or they should clearly relate it to stimulated emission that is discussed later in the paper.

This is an important nuance. We would like to point out the difference between the measured and simulated averaged charge states. In single nanoparticle experiments, we don´t have direct experimental access to the charge states at the end of the FEL pulse on the femtosecond time scale as ion detection occurs after hundreds of nanosecond times-of-flight. We have added a clarification on page 6:

"…The charge state distribution detection occurs hundreds of nanoseconds after the FEL exposure. During the sample expansion significant cooling of the plasma occurs, which leads to a variety of recombination processes lowering the overall charge states. However, previous studies indicate that the expansion velocity and the resulting recombination processes are directly correlated to the total absorbed energy during the exposure [18, 19, 51]…"

• There is a typo for "resonances" in the caption of Fig.3. *Done*

Next, I found several issues with the Supplementary section.

1) The main issue is that currently the Theory and Experimental Details sections seem to be written in a non consistent way. To be clear, I mean that quantities that appear

first in the Theory section are never defined in this section but then are defined later in the Experimental Details. The authors need to write consistently these sections so that quantities are defined already in the Theory section which is what one reads first. For instance, n_{a} is not defined in Theory but is defined in the Experiment part, f^{0}(\omega) is not discussed at all in Theory but shows up in Eq. 19 of the Experiment section.

We thank the referee for pointing out the ordering issue in the section "Calculation of the scattering cross-sections from diffraction patterns." In this section, the atomic number density $n_a$and the neutral atomic scattering factor $f^0(\omega)$were introduced in Eqs. (7)–(9) before being explicitly defined later in the text. While both quantities were subsequently defined, with $n_a$specified as the atomic number density of neon at the triple point and $f^0(\omega)$as the neutral atomic scattering factor, we agree that they should be defined at first occurrence for clarity. We have therefore revised the manuscript to introduce these quantities immediately after their first appearance. In addition, we now explicitly clarify that $f^0(\omega)$corresponds to the configuration-dependent atomic form factor used in the Theory section in the limit of a neutral atom and forward scattering.

2) Page 23, the authors mention after Eq. 1 a molecular dynamics algorithm which is used. They should cite this algorithm.

The molecular dynamics algorithm is a part of the MC/MD method. We have added an explicit citation to the molecular dynamics algorithm used in the simulations at the point where it is introduced in the Theory section.

3) It is not clear to me what is the difference between the "pulse fluence j_{x} and the fluence of the XFEL pulse in page 23. Can the authors explain?

We thank the referee for pointing out the terminology issue regarding $j_X(\tau, t)$. In the revised manuscript, we clarify that $j_X(\tau, t)$denotes the time-dependent pulse intensity (power per unit area) of the XFEL pulse, rather than the pulse fluence. The total fluence $F$is obtained by integrating $j_X(\tau, t)$over time and photon energy, as shown in Eq. (3). We have corrected the wording throughout the Supplementary Information to ensure dimensional consistency and avoid confusion.

4) Page 24, in Eq. 4 how do the authors find the form factors? Is there a reference?

Atomic form factors $f_j(\boldsymbol{q}, C_j)$are calculated from the electronic configurations using Hartree-Fock-Slater wavefunctions following Refs. [Ho 2016, Ho 2017].

5) Page 25, 2nd paragraph, the authors write, "the amplification of diffraction can go up to 6 orders of magnitude" when discussing Fig. 5. The authors should explain clearly in what part of the figure we can find these change of 6 orders of magnitude.

Done. We have added: "… as witnessed by the Ne 7+ curve (dark red line) near 900 eV…"

6) The authors refer to a phase shift between quasi-free and bound electrons that can possibly explain differences between theory and experiment. Can the authors explain how this shift enters (if it does) the Eqs the authors list in the Theory section? In my opinion, as it currently stands it is not clear what they mean.

We agree that the previous wording was unclear. The phase shift referred to would modify the relative phase between bound-electron and quasi-free-electron scattering amplitudes, affecting the interference term in the total form factor. In the current model, quasi-free electrons are treated in a phase-averaged approximation, and therefore this effect is not explicitly included. We have clarified this point in the revised Supplementary Information. We have added:

"…The phase shift mentioned here does not enter as an explicit parameter in the present model. Rather, it would modify the relative phase between the bound-electron form factor Fa(q, t) and the delocalized-electron contribution Fe(q, t), thereby changing the interference term in |Fc(q, t)|2. In the current implementation, quasi-free electrons are treated as uniformly distributed and phase-averaged, which may underestimate such interference effects in rapidly evolving nanoplasma potentials. Rapid charging during the pulse can dynamically reshape the cluster potential, potentially inducing additional phase shifts not fully captured in the present simulation …"

7) The authors should correct the colors in Fig. 5 and 6. In page 25, they say that "The neutral case is the black curve" but in Figure 5 it seems to be the purple curve. *Done* In Fig. 6 caption, again for neutral Ne they write "blue solid line" but it is actually the purple line. *Done*

8) Page 26, they write "In Fig. 5 we plot….from 0.05mJ to 0.6 mj". I believe it is Fig.6 and it is from 0.06 mJ.

The simulation is for a thought experiment with sub-fs X-ray pulse excitation and 0.6 mJ is correct. Figure 6 demonstrates that, theoretically, the scattering amplification effect through stimulated emission scales over at least one order of magnitude changes in X-ray fluence. Without stimulated emission 0.6 mJ would already lead to significant lowering of the scattering cross section in Ne atoms as witnessed by the dotted green line.

Concluding, I find this work to be very interesting. However, it needs to address the issues I mention above, i.e. consistent and careful writing of the supplemental material and mostly clearly stating in the introduction what is new compared to previous studies, so the reader can clearly assess the novelty of the work.
